# Contaminants from dredged sediments alter the transcriptome of Manila clam and induce shifts in microbiota composition

**DOI:** 10.1186/s12915-023-01741-9

**Published:** 2023-10-25

**Authors:** Ilaria Bernardini, Andrea Quagliariello, Luca Peruzza, Maria Elena Martino, Giulia Dalla Rovere, Silvia Iori, Davide Asnicar, Maria Ciscato, Jacopo Fabrello, Fabiana Corami, Martina Cecchetto, Elisa Giubilato, Claudio Carrer, Cinzia Bettiol, Elena Semenzin, Antonio Marcomini, Valerio Matozzo, Luca Bargelloni, Massimo Milan, Tomaso Patarnello

**Affiliations:** 1 Department of Comparative Biomedicine and Food Science, University of Padova, Viale Dell’Università 16, Agripolis, 35020 Legnaro, PD Italy; 2 Department of Biology, University of Padova, Via U. Bassi 58/B, 35131 Padua, Italy; 3 Aquatic Bioscience, Huntsman Marine Science Centre, 1 Lower Campus Road, E5B 2L7, St Andrews, New Brunswick Canada; 4 Department of Environmental Sciences, Informatics, and Statistics, Ca’ Foscari University of Venice, Via Torino, 155, 30172 Venice-Mestre, Italy; 5 Institute of Polar Sciences, CNR-ISP, Foscari University of Venice, Campus Scientifico - CaVia Torino, 155, 30172 Venice-Mestre, Italy; 6 Thetis S.P.a. C/o laboratorio del Provveditorato Interregionale Alle Opere Pubbliche Per Il Veneto, Il Trentino Alto Adige E Il Friuli Venezia Giulia, Venice-Mestre, Italy; 7 NFBC, National Future Biodiversity Center, Palermo, Italy

**Keywords:** Manila clam, Dredged sediments, Venice lagoon, Molecular changes, Microbiota, Lagoon contamination

## Abstract

**Background:**

The reuse of dredged sediments in ports and lagoons is a big issue as it should not affect the quality and the equilibrium of ecosystems. In the lagoon of Venice, sediment management is of crucial importance as sediments are often utilized to built-up structures necessary to limit erosion. However, the impact of sediment reuse on organisms inhabiting this delicate area is poorly known. The Manila clam is a filter-feeding species of high economic and ecological value for the Venice lagoon experiencing a drastic decline in the last decades. In order to define the molecular mechanisms behind sediment toxicity, we exposed clams to sediments sampled from different sites within one of the Venice lagoon navigable canals close to the industrial area. Moreover, we investigated the impacts of dredged sediments on clam’s microbial communities.

**Results:**

Concentrations of the trace elements and organic chemicals showed increasing concentrations from the city of Venice to sites close to the industrial area of Porto Marghera, where PCDD/Fs and PCBs concentrations were up to 120 times higher than the southern lagoon. While bioaccumulation of organic contaminants of industrial origin reflected sediments’ chemical concentrations, metal bioaccumulation was not consistent with metal concentrations measured in sediments probably due to the activation of ABC transporters. At the transcriptional level, we found a persistent activation of the mTORC1 signalling pathway, which is central in the coordination of cellular responses to chemical stress. Microbiota characterization showed the over-representation of potential opportunistic pathogens following exposure to the most contaminated sediments, leading to host immune response activation. Despite the limited acquisition of new microbial species from sediments, the latter play an important role in shaping Manila clam microbial communities.

**Conclusions:**

Sediment management in the Venice lagoon will increase in the next years to maintain and create new canals as well as to allow the operation of the new mobile gates at the three Venice lagoon inlets. Our data reveal important transcriptional and microbial changes of Manila clams after exposure to sediments, therefore reuse of dredged sediments represents a potential risk for the conservation of this species and possibly for other organisms inhabiting the Venice lagoon.

**Supplementary Information:**

The online version contains supplementary material available at 10.1186/s12915-023-01741-9.

## Background

Dredging and reuse of dredged sediments is a very intense activity in highly anthropized lagoons. However, dredged sediments represent a potential source of stressors for populations inhabiting lagoon environments. In this respect, the Venice lagoon is an emblematic case study as local authorities mobilize about 1 million m^3^/year of sediments (composed of sand, silt, and clay of both alluvial and marine origin) for navigation purposes. The physical removal of the substrate from the seabed and the resuspension and deposition of chemicals derived from industrial, agricultural and urban activities accumulated in sediments over decades are known to increase turbidity and pollutant levels in the water column with potential impacts on benthic communities [1–3].

Furthermore, the recent construction and maintenance of the MOSE system (Experimental Electromechanical Module) to protect Venice and its lagoon from flooding has significantly increased dredging activities at the three inlets of the Venice lagoon. On top of that, the Port Authority is planning to create and/or enlarge existent canals to allow the passage of large cruise ships [3–5]).

The Manila clam *Ruditapes philippinarum*, a filter-feeding bivalve inhabiting sandy-mud bottoms, represents a species potentially affected by the reuse of contaminated sediments in the Venice lagoon. Native to the Indo-Pacific region, due to its high commercial value it was introduced for farming purposes in the Mediterranean and Atlantic coasts in the early 70s [6]. Although the high adaptability and resistance to various stressors have allowed the Manila clam to spread rapidly in this area*,* overfishing, climate change and anthropogenic disturbances have been severely threatening wild and farmed populations. The lack of natural seed and increasingly frequent mortality events have significantly impacted natural populations and reduced the annual production in the Venice lagoon from 40,000 tons in 2000 to 3000 tons produced in 2019 [7]. The chemical composition of Venice lagoon sediments has been extensively studied in the past indicating that sediments stored in industrial canals are that of the most likely sources of chemical contamination to the lagoon environment [8–10]. Among the most important chemicals, high heavy metals concentrations (up to 132, 70, 48, 929 and 8295 μg g ^−1^ for As, Cd, Hg, Pb and Zn, respectively) and PAHs (up to 16,474 μg kg^ −1^) were recently described in sediments collected close the industrial area of Porto Marghera [9, 11].

However, the effect on the physiology of bivalves of their reuse remains elusive. Furthermore, changes induced by these mixtures of chemicals on the microbial community, a widely recognized fundamental marker of the animal’s well-being, are still uncharacterised and unknown. Considering the crucial importance to define the most important environmental stressors affecting lagoon environments and the adaptation strategies adopted by threatened species [12–14], in this work we assessed the responses of Manila clams exposed to sediments collected at different sites within a large navigable canal that connects the industrial area of Porto Marghera to the city of Venice. Sediment chemical analyses, bioaccumulation and whole gene expression profiles performed at different times of exposure to contaminated sediments allowed to characterize the main molecular mechanisms that Manila clams adopt to cope with the complex chemical mixture contained in sampled sediments. In addition, possible risks associated to the transfer of microbial species from sediments to biota were also explored. Overall, our findings allowed to define for the first time the risks for Manila clam populations associated to the reuse of dredged sediments.

## Results

### Sediment chemical characterization

**Fig. 1:**
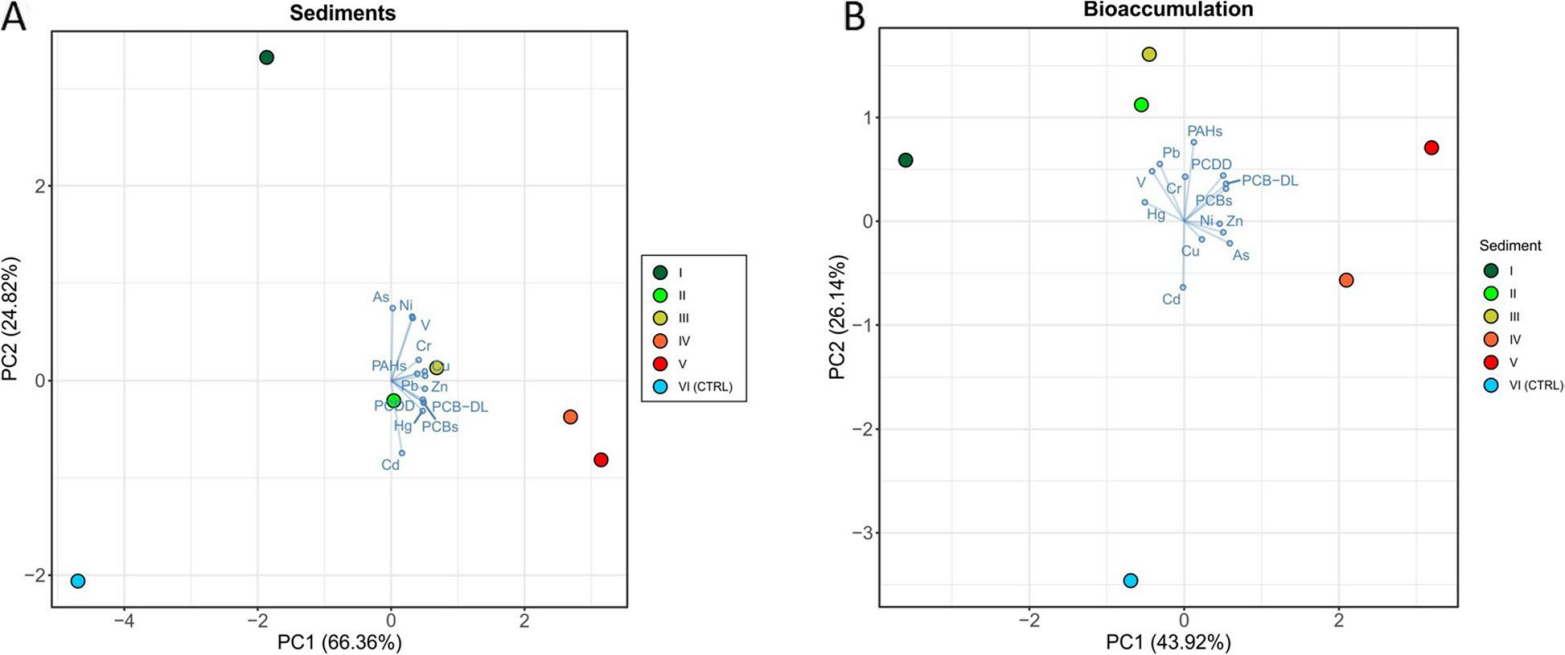
PCA considering chemical concentrations detected in sediments collected in different sites (**A**) and bioaccumulation in clams exposed to sediments collected in different sites (**B**). Different sites are indicated with different colours (green: site I; light green: site II; yellow: site III; orange: site IV; red: site V; light blue: control site). In both charts, blue arrows indicate the loadings of each variable in the PCA

Data on sediment chemical characterization are reported in Additional file 1 (Table S1 and Table S2). The highest percentage of organic matter was found in sediments collected near Porto Marghera (> 3% in sites IV and V), while the lowest was found close to the City of Venice (site I; 1.22%) and the control site (VI; < 0.3%). The sediment of the control site (VI) was mainly composed of sand (90.1%), while the silt component was predominant (> 59%) in sites I to V. The total concentration of the trace elements and organic chemicals showed similar trends for Zn, Pb, Hg, Cu, PCDD/Fs, DL-PCBs, PCBs and heavy hydrocarbons, with increasing concentrations from site I (near the city of Venice) to site V (Porto Marghera), while the control site VI revealed considerably lower concentrations for all tested chemicals (Table S2). This gradient along the Vittorio Emanuele III Canal was further confirmed by PCA analysis on chemicals detected in sediments (Fig. 1A). Sediments from different sites were clearly separated along the *x*-axis (66.36%) perfectly resembling the geographical sampling location within the canal as well as demonstrating the clear separation of the control site.

The concentrations of PCDD/Fs and PCB in sites IV and V were 72 and 120 times higher than in the control site (VI), while the concentrations of Zn, Cd and Hg in site V were 3.5, 10.7 and 50.6 times higher than in site I, respectively. The highest concentrations of PAHs were detected at site II, the closest to Venice harbour. Overall, PAHs concentrations within the five Vittorio Emanuele III sites investigated were from 63 to 148 times higher than in the control site VI. In conclusion, the concentrations of Ni and V, which are mainly lithogenic elements, were similar along the Vittorio Emanuele III Canal. The metal concentrations measured in the sediments on day 14 (end of clam exposure; Table S2B) did not show significant changes compared to T0.

### Bioaccumulation of metals and organic pollutants in clams

The bioaccumulation of metals and organic pollutants is reported in Additional file 1 (Table S3) and summarized by PCA in Fig. 1B. While clams showed slight bioaccumulation of metals, more significant bioaccumulation of PCBs, PCDD/Fs and PCB-DL was observed in clams exposed to sediments collected from site II to site V, with increasing concentrations from site II to site V. Also, WHO-TEQ values for PCDD/Fs showed the highest value in clams exposed to sediments collected from sites close to the Porto Margera industrial area (IV and V). For PAHs, we observed a similar bioaccumulation in clams exposed to sediments collected in the different sites of the Vittorio Emanuele III Canal. All the other organic pollutants studied were not detectable in the clams' tissues. Spearman’s correlation coefficients were calculated to investigate the statistical relationship between contaminants in sediments and soft tissues of clams (Table 1). A significant positive correlation was observed for PCDD/Fs, PCBs and PCB-DL. Conversely, a significant negative correlation was observed for Hg. In detail, although the Hg concentration in sediments collected from industrial sites (IV and V) was more than 45 times higher than in site I, bioaccumulation showed an opposite trend, with Hg bioaccumulation in clams exposed to sediments from sites IV and V being 82 and 43 times lower, respectively, than in T0 clams (collected from a clean farming site) and in site I clams. Similarly, higher Cd bioaccumulation was found in clams exposed to sediments from site VI (CTRL site), while clams exposed to site V sediments (which had the highest sediment Cd concentrations) showed Cd bioaccumulation similar to clams exposed to other sediments. PCA (Fig. 1B) shows the separation among treatments along the *x*-axis (43.92%), indicating organic chemicals as the main drivers of the separation of clams exposed to sediments collected along the Vittorio Emanuele III Canal from those of the control site.

**Table 1:** Spearman’s correlation coefficients (*p-value < 0.05; **p-value < 0.01; ***p-value < 0.001) calculated between contaminants in sediments (from 6 different sites) and soft clam tissues

	***R***	***p***
**V**	− 0.03	0.957
**Cr**	0.49	0.329
**Ni**	− 0.41	0.425
**Cu**	0.20	0.704
**Zn**	0.37	0.468
**As**	− 0.03	0.957
**Cd**	0.49	0.329
**Hg**	− 0.89	0.019*
**Pb**	− 0.14	0.787
**ΣPCDD/Fs**	1.00	< .001***
**ΣPCB-DL**	0.94	0.005**
**ΣPCB**	0.89	0.019*
**ΣPAHs**	0.12	0.827

### Transcriptomic analysis

**Fig. 2:**
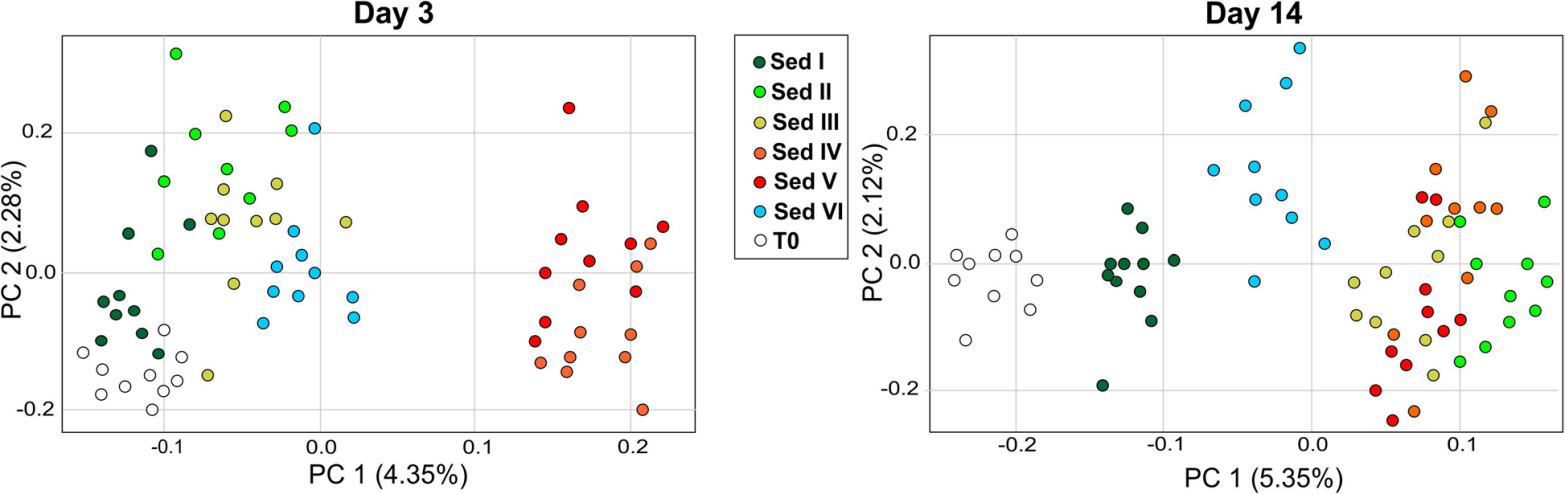
PCA considering gene expression data. PCA was obtained considering the gene expression profile of Manila clam on day 3 and day 14 (10 biological replicates for each site/sampling time). In both charts, T0 samples were also included. Colours indicate different sites (from site I to site VI)

Principal Component Analysis (PCA) performed on all samples showed a clear separation between samples collected on day 3 and day 14 (data not shown). Accordingly, we performed PCA within each sampling time (Fig. 2). After 3 days, we observed a separation along the *x*-axis (4.35%) of samples exposed to sediments collected at sites IV and V, while clams exposed to sediment I were grouped with clams at T0. After 14 days of exposure, we observed 4 main clusters along the *x*-axis (5.35%) represented by (i) pre-exposure clams (T0); (ii) clams exposed to sediment collected at site I; (iii) control clams (sediment VI); and (iv) clams exposed to sediments II, III, IV and V.

**Fig. 3:**
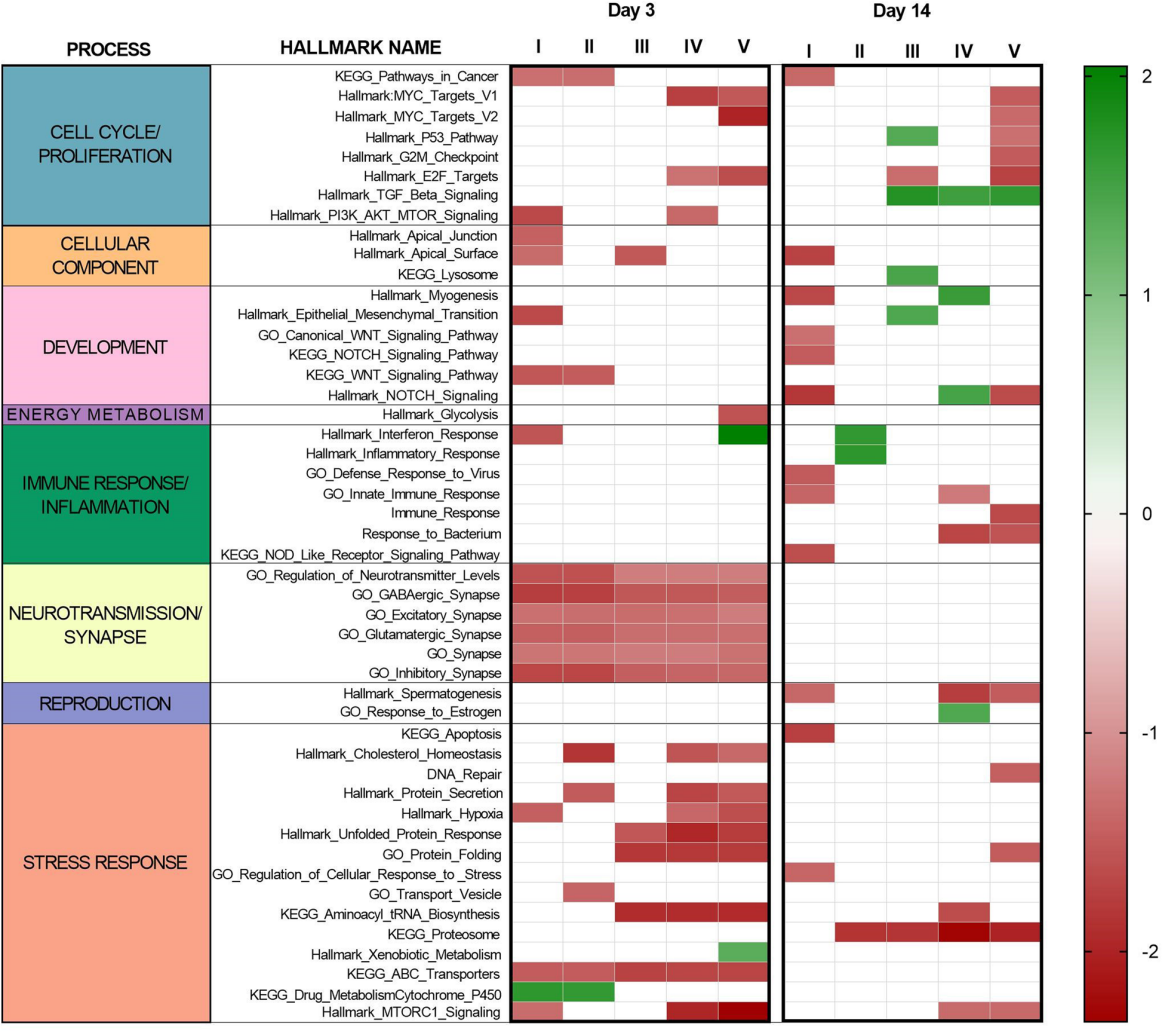
Summary of GSEA results. GSEA was obtained on day 3 and day 14 by comparing gene expression profiles of clams exposed to sediments collected in Vittorio Emanuele III (sites I, II, III, IV, V; 10 biological replicates for each site/sampling time) with control site (VI). Red and green squares indicate up- and down-regulated pathways, respectively. Red and green colour intensity is based on the corresponding normalized enrichment score (NES)

In light of the fact that we had a single replicate value for sediment chemical concentration and for bioaccumulation (i.e. one pool was analysed for each condition to reach the minimum weight required for organic chemical characterisation), while we had ten replicate data for gene expression and microbiota analyses at two sampling times, it was not possible to correlate gene expression (and microbiota) data with sediment chemicals concentration/bioaccumulation. This would have led to an important bias from a statistical point of view. To overcome this limit and to assess relationships between chemicals and gene expression profiles, we performed Pearson’s correlation between the coordinates along the first components of variation of the sediment’s PCA (Fig. 1A) and of the gene expression’s PCA after 3 and 14 days of exposure (Fig. 2). A significant correlation was found after 3 days of exposure (*R* = 0.94; *p* = 0.016) while no significant correlation was found after 14 days of treatment (Additional file 1: Fig. S1). Pairwise comparisons between the investigated sediments (from I to V) and the control site (VI) were performed separately at 3 and 14 days. The number of differentially expressed genes (DEG) obtained from each comparison is reported in Table 2, while the full lists of DEGs and corresponding annotations are reported in Additional file 2. GSEA was also performed and reported in Additional file 3 while a summary of the main results is proposed in Fig. 3.

**Table 2:** Number of differentially expressed genes (DEGs) obtained by comparing clams exposed to sediments collected in Vittorio Emanuele III channel (sites I, II, II, IV and V; 10 biological replicates for each exposure) with clams exposed to sediments collected in control site (site VI; 10 biological replicates). For each comparison, the total number of upregulated (↑) and downregulated genes (↓) in samples of each treatment are also reported

	**Day 3**			**Day 14**		
**Sediments**	**N° Total DEGs**	**↑**	**↓**	**N° Total DEGs **	**↑**	**↓**
**Site I**	1	0	1	5	0	5
**Site II**	21	6	15	36	8	28
**Site III**	0	0	0	6	1	5
**Site IV**	282	100	182	22	16	6
**Site V**	302	144	158	19	11	8

The highest number of DEGs was found on day 3 in animals exposed to sediments from the IV and V sites, with 35.6% of the common upregulated genes. Among the most interesting transcriptional changes observed in clams exposed to sediments collected at these areas were genes involved in mTORC1 signalling (e.g. *Ras-related GTP-binding protein D*, *RAGD*; *folliculin-interacting protein 1*, *FNIP*), endosome, lysosome, vesicle trafficking and protein turnover (e.g. *transitional endoplasmic reticulum ATPase*; *lysosomal-trafficking regulator LYST*; *biogenesis of lysosome-related organelles complex 1 subunit 6*; *E3 ubiquitin-protein ligases*; *transitional endoplasmic reticulum ATPase*; *cathepsin L*; *TBC1 domain family member 15*). Major molecular changes occurring in these pathways were confirmed by GSEA revealing upregulation of the gene sets “mTORC1 signalling pathway”, “protein secretion”, “unfolded protein responses”, “protein folding”, “aminoacyl tRNA biosynthesis” and “proteasome”. The heat shock proteins (*HSP12A*, *HSP12B*), the *hypoxia upregulated protein 1* and the *large proline-rich protein BAG6*, which are presumably involved in these processes and have already been widely described in general stress responses, were also found to be differentially expressed in clams exposed to sediments IV and V. GSEA showed also the upregulation of cell cycle-related gene sets in the same groups, such as “MYC targets” and “E2F targets”. DEGs involved in this process and in the regulation of apoptosis included *programmed cell death 6-interacting protein* (*PDCD6IP*), *cAMP-responsive element-binding protein-like 2*, caspases (*CASP1*, *CASP8*) and members of the inhibitor of apoptosis family (*IAP*). While ABC transporters were upregulated in all treatments (in comparison to the control sediment), site IV and V also showed the upregulation of *sulfotransferase 1C4* (*SULT1C4*), *aquaporin 5* (*AQP-5*, site IV) and *nose resistant to fluoxetine protein 6 (NRF6)*. Furthermore, on day 3, the main transcriptional responses occurring in clams exposed to sediments collected near Porto Marghera were characterized by the disruption of several genes/pathways involved in immune responses and inflammation (e.g. “interferon response”, *tumor necrosis factor ligand superfamily member 14*, *TNF receptor-associated factor 3*, *interferon-induced protein 44-like*), cholesterol homeostasis and glycolysis.

Despite the low number of DEGs, several molecular pathways were also differentially regulated in clams exposed to site I compared to the control group, including “pathways in cancer”, “interferon response”, “PI3K/AKT/MTOR signalling” and “WNT signalling pathway”. Notably, a total of six gene sets involved in synapse and neurotransmission were upregulated on day 3 in all groups (Fig. 2).

After 14 days of exposure, the number of DEGs at sites IV and V decreased to levels similar to the other sites examined (Table 2). However, even at this time point, GSEA suggested most important transcriptional changes at sites I, IV and V. Among them, clams exposed to site V showed the upregulation of several pathways involved in proliferation and cell cycle regulation (“MYC targets”, “p53 pathway”, “G2M checkpoint” and “E2F target”), “DNA repair”, “protein folding”, “mTORC1 signalling” and immune response, while clams exposed to site I sediments showed upregulation of pathways involved in signalling and development (“WNT signalling”, “myogenesis”), “pathways in cancer”, “apoptosis”, “cellular response to stress”, and immune response (e.g. “NOD-like receptor signalling”; “innate immune response”). On day 14, sites I and V also showed the common upregulation of “NOTCH signalling pathway” and “spermatogenesis” (also at site IV).

### Microbiota analysis

Principal Coordinate Analysis (PCoA) (unweighted UniFrac metrics) on digestive gland microbial communities, highlighted a clear separation between clams exposed for 3 and 14 days (Additional file 1: Fig. S2A). This demonstrates site-independent phylogenetic distance in microbiota composition between sampling time. Conversely, no significant values were obtained when considering the microbial relative abundance (Additional file 1: Table S4). Based on this result, we estimated the Unweighted UniFrac distance separately for the two time points (day 3 and day 14), in order to assess possible intra-site differences (Additional file 1: Fig. S2B). After 3 days, we obtained a clear separation of T0 samples from the remaining dataset, while all other sites showed high phylogenetic homogeneity except the control site (VI) (Additional file 1: Table S4B). Similar to the PCA performed on gene expression data, the PCoA on day 14 reported three main clusters represented by (i) T0 samples; (ii) site I and site VI samples; and (iii) sites II, III, IV and V that appear homogenous among themselves (Additional file 1: Fig. S2B). Conversely, Unweighted PCoA considering sediments microbial community shows a clear separation between sites reflecting along the *x*-axis (31%) the geographical distances between sites (Additional file 1: Fig. S2C).

The distance of clams sampled after 14 days of exposure from T0 and day 3 is mainly related to the higher abundance of ASVs belonging to Firmicutes (Additional file 1: Fig. S3). All alpha diversity metrics (i.e. richness, Shannon and Simpson indices) applied to digestive gland (DG) microbiota showed no significant diversity between sites at both sampling times, while sediment exposure resulted in a slight increase in richness of DG microbiota from T0 to day 14 (*p*-value = 0.06; Additional file 1: Fig. S4). Conversely, alpha diversity metrics applied to sediment microbiota showed significant differences between sites, with site I (near Venice) and site VI (control) having the highest and lowest diversity, respectively.

**Fig. 4:**
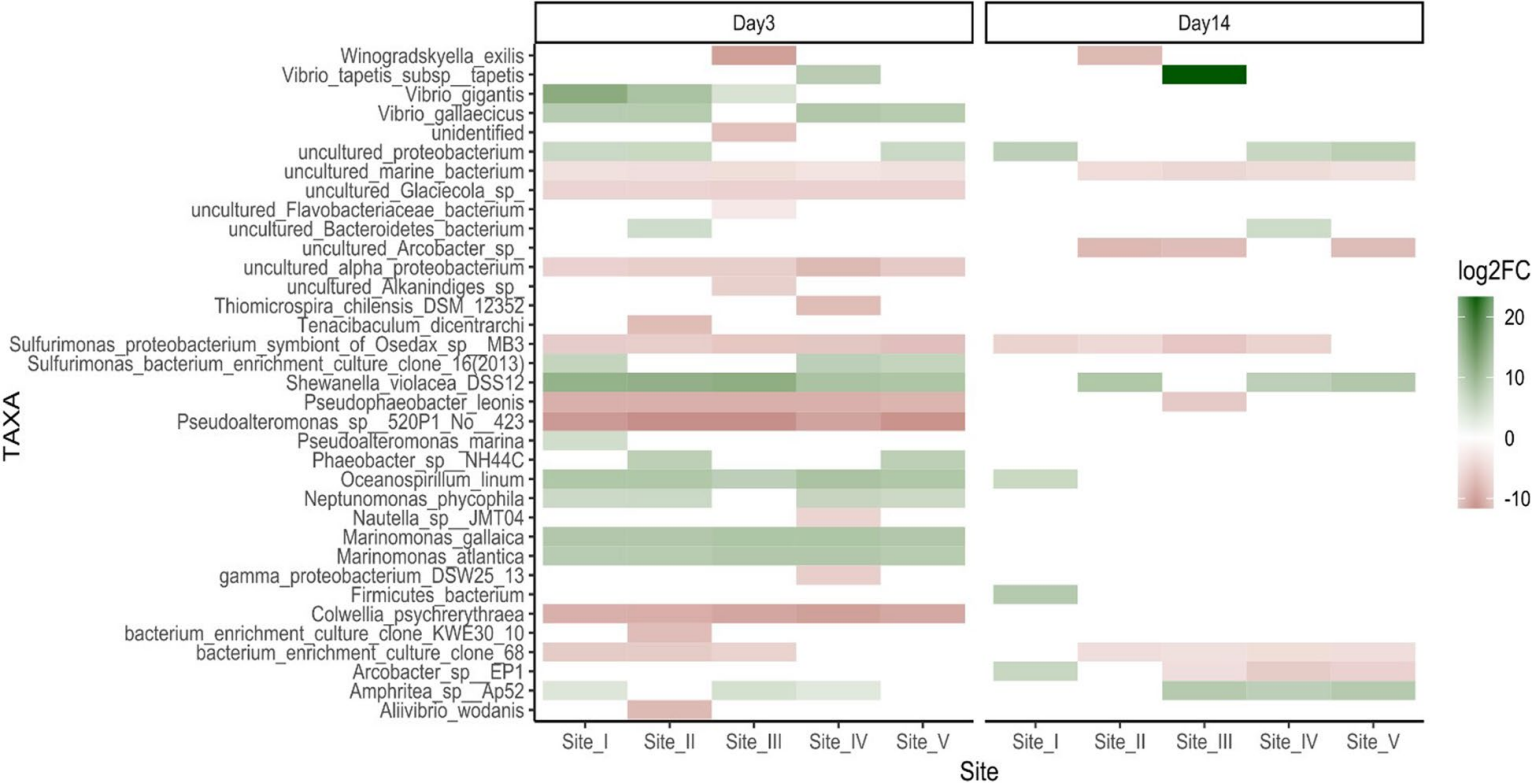
DESeq2 results at the species level for each site (10 biological replicates for each site) at the two time-points. Colour gradient represents the log2FC obtained by DESeq analysis, green boxes indicate taxa enriched in site VI, while red boxes represent taxa enriched in sites from I to V

Pairwise comparisons between the investigated sites (from I to V) and the control site (VI) for both DG (on day 3 and day 14, separately) and sediments are reported in Additional file 4 and summarized in Fig. 4. All pairwise comparisons performed considering DG microbiota showed fewer changes in DG microbiota composition on day 14 (from 5 to 8 taxa at species level) than on day 3 (from 16 to 21 taxa at species level), while the highest numbers of differentially represented taxa were found in pairwise comparisons performed between sediments microbial communities (from 35 to 47 taxa at species level). Interestingly, some species were particularly enriched in the digestive gland on day 3 in all sites compared to the control group, such as *Sulfurimonas proteobacterium symbiont of Osedax*, *Pseudophaeobacter leonis*, *Pseudoalteromonas* spp. *520P1 No 423* and *Colwellia_psychrerythraea*. On the other hand, a down-representation of *Vibrio* spp., in both DG and sediments of sites I, II, IV and V when compared to the control site was observed (Fig. 4). Although *Arcobacter* spp. was underrepresented in the sediments’ microbiota of all investigated sites, it was overrepresented on day 14 in the DG of clams exposed to sediments collected at sites III, IV and V. *Sulfurimonas* spp. also remained stably enriched on day 14 in most sites. The over-representation of the genus *Tenacibaculum* in the DG of clams exposed to sediments collected at Sites II (day 3), I and V (day 14) should also be highlighted.

Overall, from 42% (site II) to 60% (site V) of the over- or under-represented taxa in the DG on day 3 were also differentially represented in the corresponding sediment microbiota, while on day 14, the percentages ranged from 33% (site II) to 75% (site IV). Among the significant taxa obtained by comparing the sediment microbiota, from 70 to 82% were differentially represented exclusively in the sediments, with no significant changes in the DG microbial communities (data not shown). To better assess the influence of the sediment microbiota on the microbial composition of the DG, we performed for each site a pairwise comparison between T0 (pre-exposure) and day 3 + day 14 DG microbiota, identifying which of the significant taxa (ASV) obtained were also described in the corresponding sediment. Specifically, we defined two groups: (I) taxa not present in the DG microbiota at T0 and acquired from the sediments during exposure; (II) taxa overrepresented on day 3 and day 14 compared to T0 and not represented in the corresponding sediment. We found a total of 27 ASVs belonging to group I, and 10 belonging to group II (Additional file 5). The majority of group I ASVs were members of the phyla Proteobacteria and Espilonbacteraeota (with also some members of the phyla Firmicutes and Bacteroidetes), whereas group II ASVs were almost exclusively Proteobacteria (Additional file 1: Fig. S5). Among ASVs belonging to Group I shared across at least three treatments we identified *Sulfurimonas proteobacterium symbiont of Osedax* spp. (asv1004), *Thiomicrorhabdus* (asv1021), *Vibrio* (asv1028) and *Arcobacter* sp. *EP1 (*asv1009). We found that all the reported taxa followed the same trend across multiple sites, where they increased their abundance on day 3 and then decreased on day 14 (Additional file 1: Fig. S6).

Correlations between digestive gland microbial communities and gene expression data were also performed separately on day 3 and day 14. The results obtained are reported in Additional file 6. On day 3, we found a total of 20 significant correlations between transcripts and genus abundance (13 positive correlations and 7 negative correlations; *p*-value < 0.05; Rho > 0.65). On day 14, significant correlations significantly increased, with 771 negative correlations and 814 positive correlations. None of the significant correlations was confirmed at both sampling times.

## Discussion

### Activation of specific molecular pathways explains low metal bioaccumulation in clams

Gene expression profiling of Manila clams exposed to sediments collected at different sites along the Vittorio Emanuele III canal revealed site- and time-specific transcriptional changes. The most important changes at the transcriptional level were observed at an early stage in clams exposed to sediments collected at sites IV and V, which are characterized by higher chemical contamination. A significant positive correlation between sediment contamination and gene expression profiling on day 3 suggests that the level and type of contaminants in the sediments are the main drivers of gene expression changes and that these changes occur linearly as the chemical contamination increases. Conversely, the lack of significant positive correlation on day 14, as well as the lower number of DEGs at this sampling time, suggest that the activation of specific molecular pathways at an early stage allows clams to mitigate the impact of contaminants on cellular homeostasis.

Chemical analyses showed increasing concentrations of PCDD/Fs, PCBs, PCB-DL, Cd, Hg, Pb, Zn and Cu from site I to site V, suggesting their industrial origin from Porto Marghera [11, 12, 15]. However, statistical relationships between sediment contamination and bioaccumulation in clams showed significant positive correlations only for dioxins and PCBs, while no significant positive correlations were found for metals, which were detected at similar concentrations in clams exposed to different sediments. The case of Hg is emblematic, showing a significant negative correlation between sediments and bioaccumulation. These results indicate that bioaccumulation is not always consistent with metal concentrations detected in sediments, confirming the difficulty of relating metal bioaccumulation to environmental concentrations [16] as already highlighted in clams inhabiting heavily contaminated Venice lagoon sites [17]. While the lack of direct correlation between sediment contamination and bioaccumulation in natural populations may be explained by site-specific conditions (e.g. chemical-physical parameters and/or food availability affecting chemicals bioavailability and filtration rates [15, 17]), our study, conducted under controlled conditions, suggests that clams may activate molecular mechanisms capable of significantly reduce metal bioaccumulation. The ABC transporter pathway, upregulated on day 3 in clams exposed to sediments collected in the canal Vittorio Emanuele III, may play a key role in the response to metal contamination. The upregulation of this pathway was particularly evident in clams exposed to sediments collected at sites IV and V, with a total of 7 and 8 upregulated genes coding for ATP-binding cassette transporters, respectively. ABC transporters are a family of proteins that play a key role in cellular detoxification, facilitating the transport of various substances out of cells. Several studies showed that the ABC protein family can be involved in the excretion of metal ions [18–21]. Shi and Xiang [22] showed that Cu bioaccumulation in *Crassostrea angulata* was significantly higher after interference with an ABC gene, concluding that the expression levels of this gene affect the accumulation of copper in oysters. Similarly, exposure of the piscine cell line PLHC-1 to Hg leads to modulation of several ABC transporters at both transcriptional and functional levels. Although the upregulation of ABC genes was not prolonged (72 h), it resulted in persistently higher multidrug resistance protein transport activities suggesting the presence of an active involvement of efflux pumps in Hg clearance [23]. Early activation of detoxification mechanisms in clams exposed to sediments collected near Porto Marghera was also suggested by the upregulation of *AQP-5*, which belongs to a family of integral membrane proteins that function as water-selective channels with an important role in cellular osmotic balanced volume regulation [24–26]. Increased expression of AQP-encoding genes has already been described in clams inhabiting the polluted area of Porto Marghera [27], as well as under copper and lead exposure, suggesting a role in preventing the deleterious effects of metals on the cellular ion and volume regulation [21, 28].

### mTORC1 signalling plays a central role in Manila clam response to chemical stress

In our experiment, the upregulation of the mTORC1 signalling pathway and several transcriptional changes in its downstream pathways following the exposure to the most polluted sediments suggested that this pathway may play a pivotal role in the response to chemical stress. In general, mTORC1 signalling plays a central role in eukaryotic cells and organismal physiology by coordinating cell growth and metabolism with environmental conditions [29]. It senses growth signals, nutrient availability, energy status and stressors (e.g. energetic/metabolic stress, genotoxic and oxidative stress) and regulates many fundamental cellular processes, from protein synthesis to protein folding, proteome homeostasis and autophagy [30]. While several signals such as amino acid availability, insulin-like growth factor and ATP levels consistently stimulate mTORC1 activation, environmental stressors can have complex effects on mTORC1. Although mTORC1 activation by organic chemicals or heavy metals has not been extensively documented in the literature, a previous study described increased mTOR protein levels in the digestive gland of mussels exposed to benzo[a]pyrene (B[a]P) [31], as well as upregulation of a subset of genes belonging to this signalling pathway in zebrafish exposed to PCB [32] and in PCB-tolerant killifish population [33]. However, research on mTOR signalling functions in bivalve species is still limited and much remains to be explored to decipher its role in stress [34].

In our study, the potential role of mTORC1 signalling in promoting protein synthesis [29] after chemical stress is suggested by the upregulation of pathways involved in “protein secretion”, “protein folding” and “tRNA biosynthesis” observed in clams exposed to IV and V sediments. However, the concurrent activation of the “proteasome” pathway and the upregulation of several genes involved in protein degradation seem contradictory, given the fact that mTORC1 inhibits protein degradation and cellular catabolism by repressing the autophagic pathway [35–37]. The concomitant upregulation of genes involved in protein degradation and mTORC1 signalling can be explained in two possible ways, not necessarily mutually exclusive. First, increased degradation of damaged proteins following chemical exposure [38–40] may increase the availability of amino acids, a key signal to mTORC1 that the cell has sufficient nutrients for protein synthesis and cell growth. Upregulation of the Rag GTPases *RAGD* and *FNIP*, which are known to play a critical role in regulating mTORC1 activity by sensing amino acid availability, supports this hypothesis [41–44]. Overall, this may represent a mechanism capable of rapidly replacing damaged cellular components following exposure to high chemical stress. A second possibility to explain the increased proteasome activity is related to the high rates of protein synthesis (due to the activation of mTORC1 signalling) followed by an overload of endoplasmic reticulum proteins and/or a drastic reduction in translational fidelity with the synthesis of aberrant proteins, which, if not removed, may represent an additional source of cellular stress [45]. This hypothesis is partially supported by a study proposed by Yun et al. [46], which describes a mechanism by which mTORC1 couples increased protein synthesis with immunoproteasome biogenesis to protect cells from protein stress. Among others, we found upregulation of *BAG6*, a protein belonging to a cytosolic protein quality control complex involved in pre-emptive quality control induced by endoplasmic reticulum (ER) stress, which redirects newly synthesized proteins to the cytosol for protein degradation, thus protecting the ER from protein overload upon stress [47].

The inhibition of apoptosis, suggested by the downregulation of *caspase*s and upregulation of *IAP*s and *PDCD6IP* (in clams exposed to sediments IV and V on day 3), may also be partly related to mTORC1 signalling. Although the role of mTORC1 in apoptosis regulation is not universally inhibitory, recent studies have suggested that, among its downstream signalling pathways, mTORC1 can activate pro-survival signals that protect cells from apoptosis [48–50].

Upregulation of mTORC1 signalling may also play a role in other biological processes and molecular pathways, such as vesicle and protein trafficking, glycolysis and cell cycle regulation, which were found to be differentially regulated in clams exposed to sediments IV and V. The upregulation of several genes involved in endosome and vesicle trafficking may be closely related to increased protein secretion and protein turnover. Recent evidence indicates that mTORC1 directly controls Golgi architecture, endosome and lysosome distribution, and extracellular vesicle secretion [51]. In our study, among several disrupted genes involved in the transport of molecules from the trans-Golgi network to the plasma membrane, there were also the small *GTPases RAB* that are key regulators of intracellular membrane trafficking, from the formation of transport vesicles to their fusion with membranes*.* Among them, *RAB11B* represents an evolutionarily conserved subfamily of Rab GTPases involved in regulating vesicular trafficking by recycling the endosomal compartment and early endosomes to the trans-Golgi network and the plasma membrane [52–54].

mTOR signalling is also recognized as an evolutionarily conserved central regulator of cell cycle progression [55–58]. In our study, clams exposed to most contaminated sediments collected at site V showed upregulation of several pathways involved in cell cycle progression. Among them “MYC targets 1”, “MYC target _2” and “E2F targets” were upregulated at both sampling times, while “p53 pathway” and “G2M checkpoint” were upregulated at 14 days. Cross-talk between mTORC1 signalling and MYC has been widely demonstrated [59–61]. In turn, MYC which is considered to be one of the downstream targets of mTORC1 signalling, is known to regulate the expression of E2F family members, which play an important role during the G1/S transition in mammalian and plant cell cycle [62–66].

### Common and specific molecular responses to different chemicals contained in sediments

Clams exposed to sediments with the highest chemical contamination of industrial origin showed additional stress responses, mainly at an early stage. Among them, the upregulation of genes putatively involved in xenobiotic metabolism, such as SULT2 and NRF6, have already been widely described in clams populating sites close to Porto Marghera [27, 38, 40, 67]. While SULT2 belongs to a family of phase II detoxification enzymes involved in protection against xenobiotics [68–70], NRF6 was found to be positively correlated with increased concentrations of PCDD/Fs and PCB-DL in Manila clams inhabiting different contaminated sites within the Venice lagoon [40]. This confirms its potential use as a biomarker of exposure to persistent organic pollutants.

Clams exposed to sediment V also showed possible oxidative stress and DNA damage, as indicated by the upregulation of the “DNA repair pathway” and the “G2-M checkpoint” after 14 days. When DNA damage occurs, cells can activate the DNA damage checkpoint to arrest cell cycle and ensure the possibility of DNA repair [71]. The G2/M checkpoint can delay the onset of mitosis in response to a variety of environmental stressors including chemicals [72, 73] and serves as final DNA damage checkpoint before cell division. Downregulation of *citrate synthase* (CS) and the upregulation of *alternative oxidase* (AOX) in clams exposed to site V sediment on day 3 should be also highlighted. CS, which is responsible for catalysing the first reaction of the citric acid cycle, has already been proposed as a biomarker of chemical stress in bivalve species [74]. The expression of AOX is influenced by several stressors including reactive oxygen species, and may enhance the ability to resist stress reducing the level of oxidative stress induced by over-reduced electron carriers. The presence of the AOX gene has been described in many invertebrates [75, 76], and a possible role in maintaining the redox balance minimizing ROS production at the respiratory chain level has recently been proposed in *Crassostrea gigas* [77]. Disruption of “cholesterol homeostasis” was also observed after exposure to sediments collected at sites II, IV and V. While disruption of this molecular pathway in clams exposed to sediments from sites IV and V may be related to high concentrations of PCDDs and PCB [78], the disruption in clams exposed to sediments II (the closest to Venice harbour) could be due to the highest concentrations of PAHs and/or Phenanthrene detected in these sediments, as recently suggested in zebrafish embryos [79].

Despite the low number of DEGs identified at both sampling times following exposure to sediments collected near the city of Venice (site I), GSEA indicated several site-specific transcriptional changes. These included the upregulation of “pathways in cancer” and “WNT signalling pathway” at both sampling times and of “NOTCH signalling”, “apoptosis” and “cellular response to stress” on day 14. Although the sediments collected at this site showed the lowest concentrations of industrial chemicals among the sediments collected in the Canal Vittorio Emanuele III, we can speculate that some molecular changes occurring in clams exposed to these sediments could be related to the presence of other chemicals not studied in the present project. Indeed, urban emissions from the city of Venice, which in some places lacks adequate wastewater treatment facilities, can lead to relevant concentrations of several contaminants such as fragrances [80, 81], agricultural products (e.g. glyphosate), drugs (e.g. Diclofenac), antibiotics and PFAS [82] among others, as recently highlighted in the monitoring plan of the Venezia 2021 project ( [83]; unpublished data). Further studies, including the quantification of other contaminants, will help to decipher specific molecular signatures associated with responses to chemicals of urban and/or industrial origin.

However, common transcriptional changes were also described at both sampling times in clams exposed to sediments collected near the city of Venice and the industrial area of Porto Marghera. Among them, on day 3 we found the common upregulation of several pathways involved in neurotransmission (also in sites II and III) and “hypoxia”, while at the later stage (day 14) we identified the disruption of several pathways involved in spermatogenesis and immune response. In particular, upregulation of several pathways involved in the immune response was observed only in clams exposed to sediments from sites I, IV and V, which also showed an overrepresentation of potential pathogens, such as *Arcobacter* (sites IV and V) and *Tenacibalum* (sites I and V).

### Microbiota changes following exposure to sediments

Over-representation of *Arcobacter* has already been described in wild clams inhabiting Porto Marghera [84], as well as in bivalves after exposure to stressful environmental conditions [81, 82, 85–87]. *Arcobacter* spp. are considered opportunistic pathogens of several marine species [88], and are often associated with unhealthy marine animals [89–91]. *Tenacibaculum* spp. is a gram-negative and motile bacterial genus belonging to the Flavobacteriaceae and includes opportunistic species often associated with mortality events [92, 93]. Although further studies are needed to elucidate the role of this genus on Manila clams, the prevalence of *Arcobacte*r and *Tenacibaculum* may be related to the impaired physiological conditions of clams exposed to highly contaminated sediments. Despite we observed the upregulation of several molecular pathways involved in immune response in the same sites showing an over-representation of these opportunistic pathogens, no genes playing a role in immune response were significantly positively correlated to these microbial genera.

While pairwise comparisons performed on transcriptional data showed major changes in clams exposed to sediments from sites IV and V, we observed a similar number of differentially represented microbial taxa between all studied sites and the control group. However, changes in the microbial community during sediment exposure also showed major changes occurring at the early stage as observed for the expression profiling. In addition, the distribution of PCoA samples after 14 days mirrored the PCA considering gene expression data.

Among the taxa enriched in almost all Vittorio Emanuele III sites compared to the control, *Sulfurimonas* sp. is commonly found in marine sediments and exerts a strong influence on the biogeochemical cycling of sulphur and nitrogen elements found in the environment [94]. They have also been found to be positively correlated with polluted sediments [95]. In our dataset, *Sulfurimonas* sp. was found to have a very peculiar pattern of abundance across the investigated sites. Indeed, we observed a strong increase of this taxa on day 3 and then a decrease in abundance on day 14 only in sites I, II and III. We can therefore hypothesize that these sediments were probably particularly enriched in the nutritional elements required by this taxon for growth and/or to compete with other microbial taxa. Interestingly, the two sites closest to Porto Marghera and the port of Venice (Site II) were uniquely lacking *Phaeobacter* spp. on day 3, which has been shown to protect against pathogenic Vibrio in aquaculture and is therefore considered a probiotic species [96, 97].

A further change in the microbial composition was observed on day 14. Such a change was not related to the abundance of taxa, but to a general differentiation at the phylogenetic level, as highlighted by the Unweighted Unifrac distance. This is probably the result of a slow adaptation process of the whole digestive gland community to the sediment condition, as well as of the ability of Manila clam to control digestive gland microbial communities.

To the best of our knowledge, this is the first time that the influence of sediment microbiota on the microbial community of the Manila clam DG has been investigated under controlled conditions. While pairwise comparisons between sediment microbiota revealed a high number of differentially represented taxa (from 41 to 86), such changes were detected almost exclusively in sediments (from 70 to 82% of significant taxa), with no apparent changes in Manila clam DG microbiota. However, 27 out of 37 ASVs (> 73%) that increased during exposures (regardless of site) were not detected in clams at T0 but were present in sediments. Almost all of these taxa showed a significant increase in relative abundance on day 3, followed by a significant decrease on day 14. Overall, these data suggest that although clams are able to “control” the spread of several taxa over-represented in the corresponding sediments, the latter plays an important role in shaping most of the changes in microbial communities following sediment exposure.

## Conclusions

This study allowed to define the Manila clam molecular mechanisms involved in the response to complex chemical mixtures characteristic of urban and industrial areas. While the mTORC1 signalling pathway plays a key role in the coordination of several molecular and biological pathways, the upregulation of ABC transporters may explain the significant metal detoxification, leading to non-consistent bioaccumulation compared to the metal concentrations detected in the sediments. Characterization of sediment and digestive gland microbiota confirmed the ability of the Manila clam to shape the composition of its own microbial communities, limiting the acquisition of microbial species from sediments. However, the microbial species present within the sediments play an important role in shaping the changes in the Manila clam microbiota. We also observed the proliferation of opportunistic pathogens, which may represent an additional threat to clams exposed to chemical stress.

Although our data suggest that the most important transcriptional and microbial changes occur at an early stage, the reuse of dredged sediments must be considered of concern for the conservation of this species, which is experiencing a drastic reduction in its natural population, especially in heavily anthropized areas such as the Venice lagoon.

## Materials and methods

### Sediment sampling and experimental plan

**Fig. 5:**
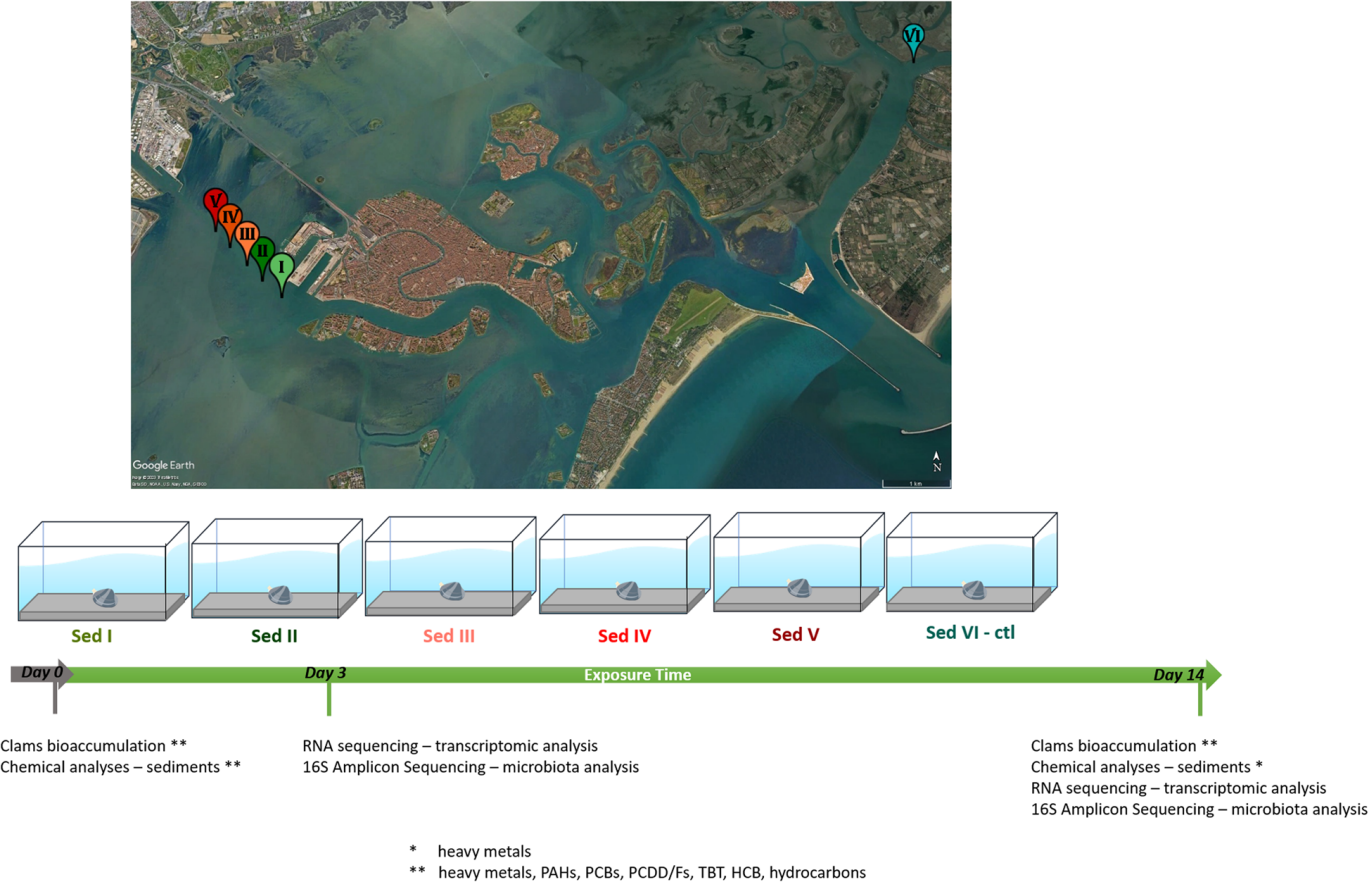
Experimental plan. Maps reported the sites where sediments were collected before transferring to the lab for homogenization. Chemical characterization, bioaccumulation, gene expression profiling and microbiota characterization are reported at the corresponding sampling time

Sediment coring was performed by SELC Company at the end of November 2020 in 5 sampling points placed in the Vittorio Emanuele III canal fairway in the Venice Lagoon (i.e. sediments I, II, III, IV and V) and one in Canale San Felice (i.e. sediment VI), which represented the control group (Fig. 5). Site I was the closest to the city of Venice, whereas site V was the closest to the industrial area of Porto Marghera hosting a large commercial port, thermo-electrical powerplants, and metallurgical and chemical companies. Site VI (reference site) was in the Canale San Felice characterized by low anthropogenic influence and classified as a high-quality site due to the fast turnover of the water [98, 99]. Four samples of sediments were collected in each sampling site using a core drill-sampling tool operated by dredge. Each core tube had a diameter of 8.55 cm and a length of 1 m. After arriving in the laboratory, the core samples from each site were extruded from the tubes, thoroughly mixed and then stored in sealed containers at – 80 °C for a few days before the experiment.

The laboratory experiment was carried out to test the responses of Manila clams to dredged sediments during their non-reproductive period. Tanks for the exposure were prepared with a sediment layer (5 cm height to guarantee the clams burrowing) and 40 L of seawater each, previously collected in an unpolluted area of the South of the Venice Lagoon, 1 day before the experiment started in order to assure the decantation of particles. Every 48 h, the seawater in the experimental tanks was changed by renewing 15 L of water.

Clams (35.1 ± 3.3 mm mean length) were collected in a farming site in the South of the Venice Lagoon and acclimated in controlled conditions for 1 week. Then a total of 50 clams were placed in each tank (each experimental group in duplicate tanks for a total of 100 clams per condition). Six different experimental groups were set in order to expose clams to sediment I (site I), II (site II), III (site III), IV (site IV), V (site V), and VI (CTL site) for a total of 14 days during which no significant mortality of animals was observed. Animals were daily fed with microalgae (*Isochrysis galbana*). Samples of sediments were collected at T0 and day 14 (from each experimental group) for chemical investigations. In addition, two samples of sediments from each tank were collected after 7 days and stored at − 80 °C for microbiota analysis. Bioaccumulation analyses were performed before (i.e. T0) and at the end of the exposure (i.e. day 14) from pools composed of approximately 10 clams' whole body. The digestive gland was sampled after 3 and 14 days of treatment from 10 individuals, and stored in RNA later at − 80 °C for molecular analyses (transcriptomic and microbiota analyses). The whole experimental plan is summarized in Fig. 3.

### Chemical analyses and sediments’ organic matter content

Determination of organic matter in the sediment was performed in triplicate following the method of Gaudette et al. [100]. Briefly, after thawing, sediments were dried, crushed by mortar and sieved on 500 μm mesh. Approximately 0.5 g of sediment was placed in a flask. The samples were added in the following order: 10 ml of potassium dichromate 1N, 20 ml of sulphuric acid 96%, the volume was increased to 200 ml with distilled water, 10 ml of phosphoric acid 85%, 5 ml of sodium fluoride and 15 drops of diphenylamine (2.5% in sulphuric acid 96%) which acts as an indicator. Titration was performed with 0.5 N ammonium ferrosulfate noting the volume of the titrant when the sample turned from black to emerald green. The result was expressed as % organic matter.

Metals concentration (i.e. V, Cr, Ni, Cu, Zn, As, Cd, Hg, Pb) in tissues of clams and in sediments (three replicates per sample) collected at T0 and day 14 were determined through the wet acid digestion with HNO_3_ (using ultrapure reagents) and H_2_O_2_ (employing a Microwave Ethos 1 Milestone and TFE vessels) and analysed by ICP-MS (ICAP Q Thermo Fisher).

Levels of organic pollutants (PCBs, PAHs, PCDD/Fs, hydrocarbons, HCB, TBT) (Chemi-lab S.r.l.) in clams were detected following the methods EPA 1668C 2010, EPA 3540C 1996 + EPA 8270E 2018, EPA 1613B 1994, RAPPORTI ISTISAN 1996/34 + EPA 8015C 2007, ICRAM 2001-SCHEDA N°7, while chemical analyses in sediments at T0 were performed by external service (Chelab s.r.l). Sediment contaminant levels and bioaccumulation among different locations were also analysed by performing a Principal Component Analysis (PCA) using the R tidymodels package. The coordinates from the PCA and the loadings were plotted using ggplot2.

### RNA extraction and sequencing

Total RNA was extracted from five digestive glands (DG) per condition per each tank (*n* = 10 tissue samples per treatment) and from two replicates of sediment from each tank (*n* = 4 sediment samples per condition in total) using RNeasy Mini Kit Qiagen (Hilden, Germany). RNA purity, concentration, and integrity were checked using a Qubit Fluorometer (Invitrogen, Carlsbad, CA, USA) and Tape Station (Agilent, Waldbronn, Germany). RNA extracted was used for both clam’s gene expression profiling (RNA-sequencing) and microbiota characterization (i.e. 16S) of clams and sediments.

Library preparation for gene expression analysis was performed using QuantSeq 3′ mRNA-Seq Library Prep Kit and the library pools were sequenced on Illumina Novaseq 6000 (single-end 75 bp) (CRIBI; University of Padova). For microbiota characterization, 1 μg of RNA was reverse-transcribed to cDNA using the Superscript IV kit (Invitrogen, Life Technologies, Monza, Italy). Libraries were prepared in a 50-μL reaction starting with diluted 0.2 ng/μL cDNA and reverse and forward primers (10 μM) specifically targeting the V3–V4 gene region of the bacterial 16S rRNA as described by Milan et al. [84]. The final libraries were then sequenced with MiSeq Illumina 300 PE (BMR Genomics, Padova, Italy). All data are deposited in NCBI (http://www.ncbi.nlm.nih.gov/genbank/ [101]; Bioproject PRJNA950925).

### Bioinformatics and statistical analyses for transcriptomic analysis

The input reads quality was assessed with FastQC/v0.11.9 [102] in order to determine and remove low-quality reads and residual adaptors by using the BBDuk program contained in the BBTools suite (program-specific options were taken from the Lexogen’s website). High-quality reads and a reference transcriptome from the digestive gland [38] were employed to perform the mapping and the count table was obtained through Kallisto/v0.46.1 [103] with default settings and finally the “abundance_estimates_to_matrix.pl” script from the Trinity suite [104]. Raw reads count were then imported into R/v3.6.0 (R Core Team 2014) and filtered: contigs with less than 5 reads in at least 20% of total libraries (out of 130 for the first) were removed to limit the background noise [105, 106]. Filtered reads were then normalized using the RUVs function (with parameter “*k* = 7”) from the RUVSeq/v1.18 library [107, 108] and then normalized counts were used to perform pairwise comparisons with edgeR/v3.26.0 [109]. A pairwise comparison was performed between the reference site VI and each experimental group within each sampling time. Genes with FDR < 0.05 and FC ≥|2| were deemed differentially expressed. Functional annotation of the reference transcriptome was performed by Blastx similarity search on Swissprot (Uniprot), *Homo sapiens* protein Ensembl database, *Danio rerio* protein Ensembl database and *Crassostrea gigas* protein Ensembl database (Evalue < 0.0001). Details and the annotation of each contig are reported in Iannello et al. [38]. Gene expression profiles were explored through the PCA as an unsupervised method performed considering all samples within each sampling time, pairwise comparisons and enrichment analyses of differentially expressed genes. Furthermore, the Gene Set Enrichment Analysis (GSEA) was performed using the Hallmark Gene Sets [110] and other gene sets reported in Additional File 3. To test the correlation between sediment data and gene expression data, the coordinates along the first component of variation of the sediment’s PCA and of the gene expression’s PCA were retrieved separately. Then Pearson’s correlation was calculated using the package “correlation” using the retrieved data. The results were then plotted using the “ggpubr” package.

### Bioinformatics and statistical analyses for microbiota analyses

Raw reads of microbiome sequencing were uploaded in QIIME 2 (Quantitative insights into microbial ecology [111] and primer sequences were removed using cutadapt. Low-quality sequences were filtered with DADA2 [112], chimeric fragments were removed and forward and reverse reads were merged to obtain high-quality representative sequences. Representative sequence alignment was performed using MAFFT software [113] and then classified using the Python library Scikit-Learn. Taxa assignment was carried out using the SILVA database (132 update release) trained for used V3-V4 primers.

Pairwise comparisons of sites from I to V against site VI were performed at genus and species level using DESeq2 [114], considering only taxa with adjusted *p*-value < 0.05. The same statistical approach was also used to compare T0 samples (before sediments exposure), against day 3 and day 14 samples separately for each site in order to highlight which taxa changed with respect to the baseline (T0). The resulting taxa were divided into two groups: group I, with taxa not detected in clams at T0 (before exposure) but over-represented on day 3 and day 14, which were also present in the respective sediment sample; group II with taxa over-represented on days 3 and 14 in digestive gland but undetected in the sediment sample. Alpha and Beta diversity analyses were computed through the phyloseq package in R software [115], as well as Adonis2 via the vegan package [116]. Spearman correlation analysis between bacteria genera (present in more than 5% of the dataset) and gene expression profiling have been also performed separately on day 3 and day 14 using the “cor” function in R (stats package). Only correlations with Rho values higher than |0.65| and with a *p*-value < 0.05 have been considered.

### Supplementary Information

**Additional file 1: Table S1.** Organic matter content and grain-size distribution in sediments collected from the six sampling sites (one replicate for each site). **Table S2.** Chemical analyses of metals and organic contaminants in sediments (one replicate for each site). **Table S3.** Bioaccumulation of metals (mg/kg dw; Table A) and organic pollutants (Table B) in clams before exposure (T0) and at the end of sediment exposure (Day 14) (one replicate for each site). **Table S4.** Pairwise Adonis values on Unweighted and Weighted Unifrac distances at different time-points (Table A) and among different sites for each time-point (Table B). Data collected from a total of 10 biological replicates for each site/sampling time were considered. **Figure S1.** Pearson’s correlation between the coordinates along the first component of variation of the sediment’s PCA and of the gene expression’s PCA. **Figure S2.** Principal Coordinate Analysis (PcoA) plot using Unweighted and Weighted UniFrac dissimilarities (ASV level) of the digestive gland and sediment microbiota. Data collected from a total of 10 biological replicates for each site/sampling time were considered. **Figure S3.** DESeq2 results by collection date. Data collected from a total of 10 biological replicates for each site/sampling time were considered. **Figure S4.** Alpha diversity in Manila clam microbiota and sediments. **Figure S5.** Barplot representing the number of unique ASVs for each bacteria Phylum in group I and group II, A and B respectively. **Figure S6.** Changes in relative abundance of the significant ASVs for the two groups and for every site considered: Site I (A); Site II (B,C); Site III (D,E); Site IV (F,G); Site V (H,I); Site VI (L,M). Green and Red lines identify ASVs belonging to group I and II, respectively.

**Additional file 2.** Lists of DEGs obtained comparing clams exposed to sediments I, II, III, IV and V with control group at Day 3 and Day 14. Pairwise comparisons were performed considering 10 biological replicate for each condition/sampling time. DEGs reported in red and green indicated up- and down- regulated genes in investigated sites, respectively. Annotation for DEGs are also reported.

**Additional file 3.** GSEA results obtained comparing clams exposed to sediments I, II, III, IV and V with control group at Day 3 and Day 14. Results are based on pairwise comparisons results performed considering 10 biological replicate for each condition/sampling time. Gene sets reported in red and green indicated up- and down- regulated pathways in investigated sites, respectively.

**Additional file 4.** Lists of significant taxa (at species and genus levels) obtained comparing clams exposed to sediments I, II, III, IV and V with control group at Day 3 and Day 14. Pairwise comparisons were performed considering 10 biological replicate (individual) for each condition/sampling time. Lists of significant taxa (at species and genus levels) obtained comparing sediments I, II, III, IV and V with control sediments are also reported. Pairwise comparisons were performed considering 3 replicates (3 sediments sampled in different tanks) for each site.

**Additional file 5.** List of significant taxa (ASV) obtained comparing T0 microbiota with microbiota at Day 3 and Day 14 for each site (10 biological replicates). Within each list we defined two groups: I) taxa not present in the DG microbiota at T0 and acquired from the sediments during exposure (green box); II) taxa overrepresented at Day 3 and Day 14 compared to T0 and not represented in the corresponding sediment (red box).

**Additional file 6.** List of microbial taxa (at genus level) and transcripts (with corresponding annotation) significantly correlated at Day 3 and Day 14. For each significant correlation rho and *p*-value are also reported. Results were obtained considering gene expression and microbiota data obtained from a total of 120 clams.

## Data Availability

All data generated during this study are included in this published article, its supplementary information files and publicly available repositories. In detail, 16S Sequence data and RNAseq data sequencing files are available in NCBI Sequence Read Archive (http://www.ncbi.nlm.nih.gov/genbank/; Bioproject PRJNA950925).
