# Supplementary material for: Contaminants from dredged sediments alter the transcriptome of Manila clam and induce shifts in microbiota composition
Source: BMC Biol. 2023 Oct 25;21:234. doi: 10.1186/s12915-023-01741-9 (PMC10601118; doi:10.1186/s12915-023-01741-9)
Supplement: Supplementary file 1 — Additional file 1: Table S1. Organic matter content and grain-size distribution in sediments collected from the six sampling sites (one replicate for each site). Table S2. Chemical analyses of metals and organic contaminants in sediments (one replicate for each site). Table S3. Bioaccumulation of metals (mg/kg dw; Table A) and organic pollutants (Table B) in clams before exposure (T0) and at the end of sediment exposure (Day 14) (one replicate for each site). Table S4. Pairwise Adonis values on Unweighted and Weighted Unifrac distances at different time-points (Table A) and among different sites for each time-point (Table B). Data collected from a total of 10 biological replicates for each site/sampling time were considered. Figure S1. Pearson’s correlation between the coordinates along the first component of variation of the sediment’s PCA and of the gene expression’s PCA. Figure S2. Principal Coordinate Analysis (PcoA) plot using Unweighted and Weighted UniFrac dissimilarities (ASV level) of the digestive gland and sediment microbiota. Data collected from a total of 10 biological replicates for each site/sampling time were considered. Figure S3. DESeq2 results by collection date. Data collected from a total of 10 biological replicates for each site/sampling time were considered. Figure S4. Alpha diversity in Manila clam microbiota and sediments. Figure S5. Barplot representing the number of unique ASVs for each bacteria Phylum in group I and group II, A and B respectively. Figure S6. Changes in relative abundance of the significant ASVs for the two groups and for every site considered: Site I (A); Site II (B,C); Site III (D,E); Site IV (F,G); Site V (H,I); Site VI (L,M). Green and Red lines identify ASVs belonging to group I and II, respectively. [file 12915_2023_1741_MOESM1_ESM.docx]

***Table S1.*** *Grain size distribution determined by laser diffraction analysis and organic matter content (mean percentage ± standard deviation) in the sediment samples from the six sampling sites collected in the two campaigns and used, respectively, for the 1st and 2nd laboratory exposure. The grain size data are reported as volume percentages in three size classes, clay (d<3.9 µm), silt (3.9 µm<d<63 µm) and sand (d>63 µm), according to the Udden-Wentworth scale.*

| **Grain size distribution** | | **Clay (%)** | **Silt (%)** | **Sand (%)** | **Organic matter (%)** |
| --- | --- | --- | --- | --- | --- |
| **Site I** |  | 19.5 | 72.6 | 7.9 | 1.22 ± 0.18 |
|  |  |  |  |  |  |
| **Site II** |  | 14.6 | 59.2 | 26.2 | 2.17 ± 0.05 |
|  |  |  |  |  |  |
| **Site III** |  | 19.0 | 71.4 | 9.6 | 2.52 ± 0.06 |
|  |  |  |  |  |  |
| **Site IV** |  | 20.3 | 69.6 | 10.1 | 3.07 ± 0.10 |
|  |  |  |  |  |  |
| **Site V** |  | 18.7 | 69.7 | 11.6 | 3.21 ± 0.10 |
|  |  |  |  |  |  |
| **Site VI** |  | 2.4 | 7.5 | 90.1 | 0.24 ± 0.04 |
|  |  |  |  |  |  |

***Table S2****. Chemical analyses of metals and organic contaminants in sediments.* ***Table A.*** *Metals concentrations detected at T0 (before transferring in tanks);* ***Table B.*** *Metals concentrations detected at Day14;* ***Table C.*** *Organic chemicals concentrations detected at T0. Measurement error of total concentration, expressed as the percentage of the relative standard deviation (RSD %) was ≤ 10%. TEQ values were calculated on the basis of 2005 WHO TEFs, using zero for the contribution of non-quantified congeners (“lower-bound” approach)*

**A.**

|  | **V** | **Cr** | **Ni** | **Cu** | **Zn** | **As** | **Cd** | **Hg** | **Pb** |
| --- | --- | --- | --- | --- | --- | --- | --- | --- | --- |
| Site I – T0 | 35.5 | 13.4 | 13.7 | 11.7 | 39.1 | 9.60 | 0.068 | 0.0183 | 14.2 |
| Site II – T0 | 24.1 | 12.1 | 9.31 | 16.8 | 77.1 | 5.20 | 0.409 | 0.535 | 20.2 |
| Site III – T0 | 26.5 | 23.4 | 10.2 | 19.1 | 80.8 | 5.03 | 0.360 | 0.624 | 22.0 |
| Site IV – T0 | 30.5 | 17.7 | 11.0 | 22.4 | 115 | 6.13 | 0.590 | 0.839 | 28.6 |
| Site V – T0 | 29.5 | 18.7 | 11.0 | 23.1 | 135 | 6.44 | 0.733 | 0.926 | 30.9 |
| Site VI (CTRL) – T0 | 9.43 | 3.77 | 2.46 | 1.11 | 6.75 | 4.52 | 0.621 | 0.105 | 2.17 |

**B.**

| **Day 14** | **V** | **Cr** | **Ni** | **Cu** | **Zn** | **As** | **Cd** | **Hg** | **Pb** |
| --- | --- | --- | --- | --- | --- | --- | --- | --- | --- |
| Site I – Day 14 | 30.6 | 14.3 | 13.7 | 11.9 | 41.9 | 11.2 | 0.0934 | 0.0168 | 15.5 |
| Site II – Day 14 | 25.0 | 12.8 | 10.5 | 18.1 | 83.4 | 5.54 | 0.407 | 0.575 | 22.2 |
| Site III – Day 14 | 26.9 | 19.4 | 10.6 | 21.6 | 91.0 | 5.12 | 0.351 | 0.648 | 24.2 |
| Site IV – Day 14 | 31.1 | 19.4 | 12.9 | 25.8 | 129 | 6.84 | 0.653 | 0.821 | 29.9 |
| Site V – Day 14 | 30.3 | 18.6 | 11.0 | 23.7 | 140 | 6.09 | 0.759 | 0.939 | 33.3 |
| Site VI (CTRL) – Day 14 | 11.6 | 3.57 | 2.93 | 1.20 | 8.77 | 4.35 | 0.648 | 0.106 | 2.86 |

**C.**

| **PCDD/Fs** | | | | | | | |
| --- | --- | --- | --- | --- | --- | --- | --- |
| **PCDD/Fs** | Units | **Site I** | **Site II** | **Site III** | **Site IV** | **Site V** | **Site VI** |
| **2,3,7,8-TetraCDD** | pg/g | <0.050 | <0.048 | 0.099 | 0.287 | 0.195 | <0.048 |
| **1,2,3,7,8-PentaCDD** | pg/g | 0.298 | 0.289 | 0.199 | <0.048 | 0.68 | <0.048 |
| **1,2,3,4,7,8-HexaCDD** | pg/g | 0.4 | 0.48 | 0.5 | 0.76 | 0.59 | <0.048 |
| **1,2,3,6,7,8-HexaCDD** | pg/g | <0.050 | <0.048 | 0.99 | 0.86 | 0.98 | <0.048 |
| **1,2,3,7,8,9-HexaCDD** | pg/g | <0.050 | 0.386 | 0.6 | 0.76 | 2.15 | <0.048 |
| **1,2,3,4,6,7,8-HeptaCDD** | pg/g | 2.28 | 9.5 | 10.6 | 19 | 24.3 | 0.48 |
| **OctaCDD** | pg/g | 8.9 | 40 | 47 | 77 | 92 | 1.34 |
| **2,3,7,8-TetraCDF** | pg/g | <0.050 | 2.7 | 3.48 | 7.1 | 9 | <0.048 |
| **1,2,3,7,8-PentaCDF** | pg/g | 0.7 | 2.03 | 4.6 | 7.6 | 11 | <0.048 |
| **2,3,4,7,8-PentaCDF** | pg/g | 0.298 | 0.96 | 3.78 | 5.2 | 7.1 | <0.048 |
| **1,2,3,4,7,8-HexaCDF** | pg/g | 0.99 | 8.8 | 13.8 | 22.7 | 32.9 | <0.048 |
| **1,2,3,6,7,8-HexaCDF** | pg/g | 1.39 | 4.4 | 7 | 11.8 | 16.8 | <0.048 |
| **1,2,3,7,8,9-HexaCDF** | pg/g | 0.5 | 1.06 | 1.09 | 5.1 | 2.44 | <0.048 |
| **2,3,4,6,7,8-HexaCDF** | pg/g | <0.050 | 1.83 | 4.1 | 6.4 | 9.2 | <0.048 |
| **1,2,3,4,6,7,8-HeptaCDF** | pg/g | 9.2 | 38.3 | 57 | 100 | 131 | 0.86 |
| **1,2,3,4,7,8,9-HeptaCDF** | pg/g | 2.18 | 6.6 | 8.7 | 13.4 | 21.6 | 0.288 |
| **OctaCDF** | pg/g | 34 | 78 | 106 | 190 | 279 | 2.3 |
| **Σ PCDD/Fs** | pg/g | **61.1** | **195** | **270** | **468** | **640.9** | **5.27** |
| **WHO-TEQ** | pg WHO-TEQ/g | **0.886** | **3.18** | **5.53** | **9.03** | **12.6** | **0.017** |
| **DL-PCBs** | | | | | | | |
| **DL-PCBs** | Units | **Site I** | **Site II** | **Site III** | **Site IV** | **Site V** | **Site VI** |
| **3,3',4,4'-Tetrachlorobiphenyl (77)** | pg/g | 9.9 | 66 | 75 | 156 | 172 | 1.92 |
| **3,4,4',5-Tetrachlorobiphenyl (81)** | pg/g | <0.67 | 1.16 | <0.67 | 1.72 | 2.34 | <0.64 |
| **2,3,3',4,4'-Pentachlorobiphenyl (105)** | pg/g | 43 | 314 | 370 | 620 | 650 | 6.5 |
| **2,3,4,4',5-Pentachlorobiphenyl (114)** | pg/g | 7.2 | 11.8 | 13.8 | 21.3 | 20.3 | <0.64 |
| **2,3',4,4',5-Pentachlorobiphenyl (118)** | pg/g | 146 | 1300 | 1450 | 2750 | 2760 | 23.3 |
| **2',3,4,4',5-Pentachlorobiphenyl (123)** | pg/g | 2.18 | 18.6 | 24.5 | 34.5 | 39 | <0.64 |
| **3,3',4,4',5-Pentachlorobiphenyl (126)** | pg/g | <0.67 | 4.9 | 5.9 | 11.3 | 11.7 | <0.64 |
| **2,3,3',4,4',5-Hexachlorobiphenyl (156)** | pg/g | 14.4 | 114 | 139 | 231 | 231 | 2.21 |
| **2,3,3',4,4',5'-Hexachlorobiphenyl (157)** | pg/g | 2.58 | 29.2 | 32.5 | 59 | 56 | <0.64 |
| **2,3',4,4',5,5'-Hexachlorobiphenyl (167)** | pg/g | 10.2 | 73 | 80 | 134 | 135 | 1.15 |
| **3,3',4,4',5,5'-Hexachlorobiphenyl (169)** | pg/g | <0.67 | <0.65 | 0.99 | 2.1 | 1.76 | <0.64 |
| **2,3,3',4,4',5,5'-Heptachlorobiphenyl (189)** | pg/g | 4.2 | 16.6 | 18.6 | 36.5 | 32.7 | <0.64 |
| **Σ DL-PCBs** | pg/g | **240** | **1949** | **2210** | **4057** | **4112** | **35.1** |
| **WHO-TEQ** | pg WHO-TEQ/g | **0.0079** | **0.553** | **0.691** | **1.33** | **1.36** | **0.0012** |
| **PCBs** | | | | | | | |
| **PCBs** | **Units** | **Site I** | **Site II** | **Site III** | **Site IV** | **Site V** | **Site VI** |
| 2-Chlorobiphenyl **(1)** | pg/g | 6.5 | 10.6 | 11.4 | 13.1 | 11.8 | 4 |
| 4-Chlorobiphenyl **(3)** | pg/g | 12.6 | 22.1 | 16.2 | 26.4 | 28.6 | 3.07 |
| 2,2'-Dichlorobiphenyl **(4)** | pg/g | 14.1 | 24.5 | 23.5 | 31.1 | 35.6 | 8.8 |
| 4,4'-Dichlorobiphenyl **(15)** | pg/g | 22.5 | 102 | 117 | 211 | 253 | 7.5 |
| 2,2',6-Trichlorobiphenyl **(19)** | pg/g | 3.28 | 12.8 | 12.3 | 16.3 | 18.4 | 2.59 |
| 2,4,4'-Trichlorobiphenyl **(28)** | pg/g | 146 | 810 | 900 | 1740 | 1840 | 60 |
| 3,4,4'-Trichlorobiphenyl **(37)** | pg/g | 23.3 | 156 | 177 | 355 | 410 | 6.8 |
| 2,2',5,5'-Tetrachlorobiphenyl **(52)** | pg/g | 91 | 760 | 700 | 1290 | 1470 | 28.3 |
| 2,2',6,6'-Tetrachlorobiphenyl **(54)** | pg/g | <0.67 | 2.51 | 2.39 | 2.29 | 1.85 | <0.64 |
| 3,3',4,4'-Tetrachlorobiphenyl **(77)** | pg/g | 9.9 | 66 | 75 | 156 | 172 | 1.92 |
| 3,4,4',5-Tetrachlorobiphenyl **(81)** | pg/g | <0.67 | 1.16 | <0.67 | 1.72 | 2.34 | <0.64 |
| 2,2',3,5',6-Pentachlorobiphenyl **(95)** | pg/g | 68 | 570 | 540 | 1030 | 1060 | 16.1 |
| 2,2',4,4',5-Pentachlorobiphenyl **(99)** | pg/g | 61 | 710 | 700 | 1420 | 1530 | 12.2 |
| 2,2',4,5,5'-Pentachlorobiphenyl **(101)** | pg/g | 91 | 890 | 920 | 1710 | 1840 | 18.5 |
| 2,2',4,6,6'-Pentachlorobiphenyl **(104)** | pg/g | <0.67 | <0.65 | <0.67 | <0.64 | <0.65 | <0.64 |
| 2,3,3',4,4'-Pentachlorobiphenyl **(105)** | pg/g | 43 | 314 | 370 | 620 | 650 | 6.5 |
| 2,3,3',4',6-Pentachlorobiphenyl **(110)** | pg/g | 73 | 740 | 730 | 1380 | 1350 | 15.6 |
| 2,3,4,4',5-Pentachlorobiphenyl **(114)** | pg/g | 7.2 | 11.8 | 13.8 | 21.3 | 20.3 | <0.64 |
| 2,3',4,4',5-Pentachlorobiphenyl **(118)** | pg/g | 146 | 1300 | 1450 | 2750 | 2760 | 23.3 |
| 2',3,4,4',5-Pentachlorobiphenyl **(123)** | pg/g | 2.18 | 18.6 | 24.5 | 34.5 | 39 | <0.64 |
| 3,3',4,4',5-Pentachlorobiphenyl **(126)** | pg/g | <0.67 | 4.9 | 5.9 | 11.3 | 11.7 | <0.64 |
| 2,2',3,3',4,4'-Hexachlorobiphenyl **(128)** | pg/g | 25 | 209 | 254 | 460 | 450 | 3.9 |
| 2,2',3,4,4',5'-Hexachlorobiphenyl **(138)** | pg/g | 112 | 970 | 1220 | 2200 | 2150 | 20.5 |
| 2,2',3,4',5,5'-Hexachlorobiphenyl **(146)** | pg/g | 34.8 | 390 | 400 | 810 | 850 | 9.6 |
| 2,2',3,4',5',6-Hexachlorobiphenyl **(149)** | pg/g | 93 | 1020 | 1020 | 1970 | 1940 | 20 |
| 2,2',3,5,5',6-Hexachlorobiphenyl **(151)** | pg/g | 28.5 | 253 | 261 | 480 | 470 | 5.3 |
| 2,2',4,4',5,5'-Hexachlorobiphenyl **(153)** | pg/g | 164 | 1680 | 1840 | 3530 | 3620 | 31.7 |
| 2,2',4,4',6,6'-Hexachlorobiphenyl **(155)** | pg/g | <0.67 | 1.06 | 0.99 | 2.87 | 3.32 | <0.64 |
| 2,3,3',4,4',5-Hexachlorobiphenyl **(156)** | pg/g | 14.4 | 114 | 139 | 231 | 231 | 2.21 |
| 2,3,3',4,4',5'-Hexachlorobiphenyl **(157)** | pg/g | 2.58 | 29.2 | 32.5 | 59 | 56 | <0.64 |
| 2,3',4,4',5,5'-Hexachlorobiphenyl **(167)** | pg/g | 10.2 | 73 | 80 | 134 | 135 | 1.15 |
| 3,3',4,4',5,5'-Hexachlorobiphenyl **(169)** | pg/g | <0.67 | <0.65 | 0.99 | 2.1 | 1.76 | <0.64 |
| 2,2',3,3',4,4',5-Heptachlorobiphenyl **(170)** | pg/g | 40 | 325 | 380 | 680 | 650 | 6.2 |
| 2,2',3,3',4,4',6-Heptachlorobiphenyl **(171)** | pg/g | 10.1 | 80 | 96 | 176 | 165 | <0.64 |
| 2,2',3,3',4',5,6-Heptachlorobiphenyl **(177)** | pg/g | 14.3 | 129 | 153 | 268 | 251 | 2.21 |
| 2,2',3,4,4',5,5'-Heptachlorobiphenyl **(180)** | pg/g | 69 | 610 | 700 | 1270 | 1170 | 9.5 |
| 2,2',3,4,4',5',6-Heptachlorobiphenyl **(183)** | pg/g | 19.7 | 155 | 169 | 325 | 302 | 3.36 |
| 2,2',3,4',5,5',6-Heptachlorobiphenyl **(187)** | pg/g | 54 | 560 | 540 | 990 | 1020 | 8.1 |
| 2,2',3,4',5,6,6'-Heptachlorobiphenyl **(188)** | pg/g | 0.79 | 10.6 | 9 | 20.1 | 19.7 | <0.64 |
| 2,3,3',4,4',5,5'-Heptachlorobiphenyl **(189)** | pg/g | 4.2 | 16.6 | 18.6 | 36.5 | 32.7 | <0.64 |
| 2,2',3,3',5,5',6,6'-Octachlorobiphenyl **(202)** | pg/g | 5.1 | 48 | 46 | 80 | 77 | <0.64 |
| 2,3,3',4,4',5,5',6-Octachlorobiphenyl **(205)** | pg/g | 7 | 9.7 | 9.2 | 16.9 | 16.6 | <0.64 |
| 2,2',3,3',4,4',5,5',6-Nonachlorobiphenyl **(206)** | pg/g | 18.8 | 46 | 52 | 94 | 115 | <0.64 |
| 2,2',3,3',4,5,5',6,6'-Nonachlorobiphenyl **(208)** | pg/g | 68 | 112 | 100 | 147 | 171 | 2.5 |
| Decachlorobiphenyl **(209)** | pg/g | 400 | 279 | 323 | 490 | 680 | 42 |
| **Σ NDL-PCBs (indicator PCBs - 6 congeners)** | pg/g | **673** | **5720** | **6280** | **11740** | **12090** | **169** |
| **Σ PCBs (regulated PCBs - 13 congeners)** | pg/g | **868** | **7415** | **8205** | **15352** | **15719** | **200** |
| **PAHs** | | | | | | | |
| **PAHs** | Units | **Site I** | **Site II** | **Site III** | **Site IV** | **Site V** | **Site VI** |
| **Naphthalene** | ng/g | 2.16 | 7.3 | 7 | 11.4 | 11.1 | <0.55 |
| **Acenaphthene** | ng/g | <0.66 | 4.04 | 2.25 | 3.13 | 2.75 | <0.65 |
| **Acenaphthylene** | ng/g | 0.99 | 2.55 | 1.83 | 4.23 | 4.12 | <0.77 |
| **Anthracene** | ng/g | 2.36 | 8.9 | 4.12 | 5.99 | 4.68 | <0.78 |
| **Benzo[a]anthracene** | ng/g | 15.7 | 36.1 | 17.5 | 28.5 | 20.3 | <1.4 |
| **Benzo[a]pyrene** | ng/g | 14.8 | 42.1 | 20.5 | 34.7 | 24 | <0.72 |
| **Benzo[b]fluoranthene** | ng/g | 18.6 | 55 | 26.6 | 63 | 29.8 | <1.6 |
| **Benzo[e]pyrene** | ng/g | 14.9 | 42.1 | 22.1 | 41.1 | 28.2 | 0.81 |
| **Benzo[g.h.i]perylene** | ng/g | 9.7 | 33.6 | 17.2 | 36 | 25 | 0.71 |
| **Benzo[j]fluorantene** | ng/g | 16.9 | 43.1 | 29.4 | 31.7 | 24 | <1.4 |
| **Benzo[k]fluoranthene** | ng/g | 7.1 | 22.6 | 13.8 | 25.4 | 12.3 | <1.3 |
| **Chrysene** | ng/g | 12.8 | 30.5 | 16.9 | 26.5 | 18.4 | <0.41 |
| **Dibenzo[a.e]pyrene** | ng/g | 3.98 | 10.3 | 6.3 | 10.7 | 7.4 | <2 |
| **Dibenzo[a.h]anthracene** | ng/g | 3.27 | 11.3 | 5 | 10.7 | 7.3 | <1.2 |
| **Dibenzo[a.h]pyrene** | ng/g | 2.3 | 4.33 | 2.54 | 3.14 | 1.86 | <0.77 |
| **Dibenzo[a.i]pyrene** | ng/g | 3.36 | 10.8 | 5.4 | 5.7 | 4.89 | <1.7 |
| **Dibenzo[a.l]pyrene** | ng/g | 7.7 | 12.2 | 3.96 | 9.1 | 4.37 | <0.74 |
| **Phenanthrene** | ng/g | 9.3 | 40.4 | 19 | 27.8 | 23.7 | <0.44 |
| **Fluoranthene** | ng/g | 33.7 | 67 | 34.5 | 51.6 | 40.3 | 1.07 |
| **Fluorene** | ng/g | 1.39 | 6.08 | 3.69 | 6.11 | 5.3 | <1.1 |
| **Indeno[1.2.3-cd]pyrene** | ng/g | 8.3 | 28.3 | 14.3 | 27.7 | 18.9 | <0.86 |
| **Perylene** | ng/g | 13 | 21.1 | 13 | 25.2 | 15.7 | <3.2 |
| **Pyrene** | ng/g | 25 | 51.1 | 29 | 43.4 | 37.1 | 1 |
| **Σ PAHs** | ng/g | **227** | **591** | **316** | **533** | **371** | **3.59** |
| **Hydrocarbons** | | | | | | | |
| **Hydrocarbons** | Units | **Site I** | **Site II** | **Site III** | **Site IV** | **Site V** | **Site VI** |
| **Light hydrocarbons ≤ C12** | mg/kg | 0.92 | 0.72 | 1.29 | 0.95 | 1.31 | <0.14 |
| **Heavy hydrocarbons > C12** | mg/kg | 13.2 | 23.4 | 30.2 | 73 | 62 | <1.6 |
| **Hexachlorobenzene** | | | | | | | |
|  | Units | **Site I** | **Site II** | **Site III** | **Site IV** | **Site V** | **Site VI** |
| **Hexachlorobenzene** | ng/g | <0.58 | <0.59 | 0.77 | 1.04 | <0.55 | <0.57 |

***Table S3****. Bioaccumulation of metals (mg/kg dw;* ***Table A****) and organic pollutants (****Table B****) in clams before exposure (T0) and at the end of sediments exposure (Day 14). TEQ values were calculated on the basis of 2005 WHO TEFs, using zero for the contribution of non-quantified congeners (“lower-bound” approach)*

**A.**

|  | **V** | **Cr** | **Ni** | **Cu** | **Zn** | **As** | **Cd** | **Hg** | **Pb** |
| --- | --- | --- | --- | --- | --- | --- | --- | --- | --- |
| **T0** | 1,05 | 1,48 | 4,31 | 4,13 | 35,8 | 9,30 | 0,212 | 0,0970 | 0,426 |
|  |  |  |  |  |  |  |  |  |  |
| **Site I – Day 14** | 0,932 | 0,628 | 2,52 | 3,05 | 30,6 | 6,87 | 0,101 | 0,0511 | 0,784 |
| **Site II – Day 14** | 1,04 | 0,923 | 5,07 | 4,09 | 31,1 | 8,03 | 0,188 | 0,0467 | 0,748 |
| **Site III – Day 14** | 0,840 | 0,942 | 3,96 | 3,49 | 38,3 | 7,49 | 0,073 | 0,00909 | 0,812 |
| **Site IV – Day 14** | 0,122 | 0,337 | 3,98 | 3,09 | 37,9 | 9,41 | 0,100 | 0,00098 | 0,247 |
| **Site V – Day 14** | 0,470 | 0,934 | 5,41 | 4,24 | 48,8 | 10,1 | 0,148 | 0,00118 | 0,638 |
| **Site VI (CTRL) – Day 14** | 0,398 | 0,638 | 4,45 | 4,14 | 39,2 | 8,62 | 0,300 | 0,0147 | 0,438 |

**B.**

| **PCDD/Fs** | | | | | | | | |
| --- | --- | --- | --- | --- | --- | --- | --- | --- |
|  | **Units** | **T0** | **Site I** | **Site II** | **Site III** | **Site IV** | **Site V** | **Site VI** |
| **2,3,7,8-TetraCDD** | pg/g | <0.2 | <0.2 | <0.2 | <0.2 | <0.2 | <0.2 | <0.2 |
| **1,2,3,7,8-PentaCDD** | pg/g | <0.2 | <0.2 | <0.2 | <0.2 | <0.2 | <0.2 | <0.2 |
| **1,2,3,4,7,8-HexaCDD** | pg/g | <0.2 | <0.2 | <0.2 | <0.2 | <0.2 | <0.2 | <0.2 |
| **1,2,3,6,7,8-HexaCDD** | pg/g | <0.2 | <0.2 | <0.2 | <0.2 | <0.2 | <0.2 | <0.2 |
| **1,2,3,7,8,9-HexaCDD** | pg/g | <0.2 | <0.2 | <0.2 | <0.2 | <0.2 | <0.2 | <0.2 |
| **1,2,3,4,6,7,8-HeptaCDD** | pg/g | 0,2 | <0.2 | 0,3 | 0,4 | 0,4 | 0,4 | <0.2 |
| **OctaCDD** | pg/g | 1,3 | 0,8 | 1,4 | 1,8 | 1,6 | 1,6 | 0,6 |
| **2,3,7,8-TetraCDF** | pg/g | 0,7 | 0,9 | 1,3 | 1,1 | 1,4 | 1,5 | 1,1 |
| **1,2,3,7,8-PentaCDF** | pg/g | 0,9 | <0.2 | 0,3 | 0,3 | 0,4 | 0,4 | <0.2 |
| **2,3,4,7,8-PentaCDF** | pg/g | <0.2 | <0.2 | 0,3 | 0,3 | 0,5 | 0,4 | <0.2 |
| **1,2,3,4,7,8-HexaCDF** | pg/g | <0.2 | <0.2 | 0,4 | 0,4 | 0,5 | 0,7 | <0.2 |
| **1,2,3,6,7,8-HexaCDF** | pg/g | <0.2 | <0.2 | 0,2 | 0,3 | 0,3 | 0,3 | <0.2 |
| **1,2,3,7,8,9-HexaCDF** | pg/g | <0.2 | <0.2 | <0.2 | <0.2 | <0.2 | <0.2 | <0.2 |
| **2,3,4,6,7,8-HexaCDF** | pg/g | <0.2 | <0.2 | 0,2 | <0.2 | 0,3 | 0,4 | <0.2 |
| **1,2,3,4,6,7,8-HeptaCDF** | pg/g | 0,6 | 0,2 | 1,5 | 1,9 | 2,2 | 2,2 | <0.2 |
| **1,2,3,4,7,8,9-HeptaCDF** | pg/g | <0.2 | <0.2 | <0.2 | 0,2 | 0,3 | 0,2 | <0.2 |
| **OctaCDF** | pg/g | 1 | 0,4 | 1,9 | 2,8 | 3,1 | 3,1 | <0.2 |
| **Σ PCDD/Fs** | **pg/g** | **4,7** | **2,3** | **7,8** | **9,5** | **11** | **11,2** | **1,7** |
| **WHO-TEQ** | **pg WHO-TEQ/g** | **0,106** | **0,092** | **0,328** | **0,305** | **0,442** | **0,451** | **0,110** |
| **DL-PCBs** | | | | | | | | |
|  | **Units** | **T0** | **Site I** | **Site II** | **Site III** | **Site IV** | **Site V** | **Site VI** |
| **3,3',4,4'-Tetrachlorobiphenyl (77)** | pg/g | 54 | 38 | 46 | 41 | 51 | 53 | 34 |
| **3,4,4',5-Tetrachlorobiphenyl (81)** | pg/g | <2 | <2 | <2 | <2 | <2 | <2 | <2 |
| **2,3,3',4,4'-Pentachlorobiphenyl (105)** | pg/g | 143 | 96 | 146 | 136 | 167 | 192 | 88 |
| **2,3,4,4',5-Pentachlorobiphenyl (114)** | pg/g | 17 | 11 | 16 | 14 | 18 | 20 | 10 |
| **2,3',4,4',5-Pentachlorobiphenyl (118)** | pg/g | 367 | 255 | 611 | 490 | 747 | 795 | 231 |
| **2',3,4,4',5-Pentachlorobiphenyl (123)** | pg/g | 8 | 5 | 9 | 8 | 10 | 13 | 5 |
| **3,3',4,4',5-Pentachlorobiphenyl (126)** | pg/g | <2 | <2 | <2 | <2 | 2 | <2 | <2 |
| **2,3,3',4,4',5-Hexachlorobiphenyl (156)** | pg/g | 81 | 53 | 72 | 70 | 78 | 90 | 51 |
| **2,3,3',4,4',5'-Hexachlorobiphenyl (157)** | pg/g | 20 | 13 | 18 | 17 | 21 | 24 | 13 |
| **2,3',4,4',5,5'-Hexachlorobiphenyl (167)** | pg/g | 37 | 23 | 35 | 33 | 39 | 39 | 23 |
| **3,3',4,4',5,5'-Hexachlorobiphenyl (169)** | pg/g | 2 | <2 | <2 | <2 | <2 | <2 | <2 |
| **2,3,3',4,4',5,5'-Heptachlorobiphenyl (189)** | pg/g | 13 | 9 | 12 | 12 | 13 | 13 | 9 |
| **Σ DL-PCBs** | **pg/g** | **742** | **503** | **965** | **821** | **1146** | **1239** | **464** |
| **WHO-TEQ** | **pg WHO-TEQ/g** | **0,086** | **0,018** | **0,032** | **0,028** | **0,238** | **0,041** | **0,016** |
| **NDL-PCBs (indicator PCBs)** | | | | | | | | |
| **NDL-PCBs (indicator PCBs)** | **Units** | **T0** | **Site I** | **Site II** | **Site III** | **Site IV** | **Site V** | **Site VI** |
| 2,4,4'-Trichlorobiphenyl **(28)** | pg/g | 208 | 154 | 278 | 242 | 340 | 371 | 150 |
| 2,2',5,5'-Tetrachlorobiphenyl **(52)** | pg/g | 325 | 234 | 780 | 560 | 810 | 810 | 206 |
| 2,2',4,5,5'-Pentachlorobiphenyl **(101)** | pg/g | 800 | 580 | 1070 | 850 | 1200 | 1200 | 770 |
| 2,2',3,4,4',5'-Hexachlorobiphenyl **(138)** | pg/g | 730 | 490 | 670 | 470 | 760 | 790 | 460 |
| 2,2',4,4',5,5'-Hexachlorobiphenyl **(153)** | pg/g | 1260 | 940 | 1380 | 1200 | 1500 | 1530 | 910 |
| 2,2',3,4,4',5,5'-Heptachlorobiphenyl **(180)** | pg/g | 980 | 660 | 830 | 870 | 860 | 880 | 700 |
| **Σ NDL-PCBs (indicator PCBs)** | **pg/g** | **4303** | **3058** | **5008** | **4192** | **5470** | **5581** | **3196** |
| **PAHs** | | | | | | | | |
| **PAHs** | **Units** | **T0** | **Site I** | **Site II** | **Site III** | **Site IV** | **Site V** | **Site VI** |
| **Naphthalene** | ng/g | 8 | 27 | 13 | 11 | 9 | 8 | 9 |
| **Acenaphthylene** | ng/g | <2 | <2 | <2 | <2 | <2 | <2 | <2 |
| **Acenaphthene** | ng/g | <2 | <2 | <2 | <2 | <2 | <2 | <2 |
| **Fluorene** | ng/g | 4 | 4 | 3 | 3 | 3 | 4 | 3 |
| **Phenanthrene** | ng/g | 18 | 17 | 12 | 15 | 13 | 15 | 13 |
| **Anthracene** | ng/g | <2 | <2 | <2 | <2 | <2 | <2 | <2 |
| **Fluoranthene** | ng/g | 6 | 12 | 12 | 12 | 13 | 15 | 6 |
| **Pyrene** | ng/g | 6 | 11 | 19 | 17 | 18 | 20 | 5 |
| **Benzo[a]anthracene** | ng/g | 2 | 2 | 4 | 4 | 4 | 4 | 2 |
| **Chrysene** | ng/g | 5 | 5 | 7 | 7 | 8 | 9 | 4 |
| **Benzo[b]fluoranthene** | ng/g | <2 | <2 | 3 | 3 | 3 | 3 | <2 |
| **Benzo[k]fluoranthene** | ng/g | <2 | <2 | <2 | <2 | <2 | <2 | <2 |
| **Benzo[j]fluorantene** | ng/g | <2 | <2 | <2 | <2 | <2 | <2 | <2 |
| **Benzo[e]pyrene** | ng/g | <2 | <2 | 3 | 3 | 3 | 3 | <2 |
| **Benzo[a]pyrene** | ng/g | <2 | <2 | <2 | 2 | <2 | <2 | <2 |
| **Perylene** | ng/g | <2 | <2 | <2 | 2 | 2 | 2 | <2 |
| **Indeno[1.2.3-cd]pyrene** | ng/g | <2 | <2 | <2 | <2 | <2 | <2 | <2 |
| **Dibenzo[a.h]anthracene** | ng/g | <2 | <2 | <2 | <2 | <2 | <2 | <2 |
| **Benzo[g.h.i]perylene** | ng/g | <2 | <2 | <2 | 2 | <2 | <2 | <2 |
| **Dibenzo[a.i]pyrene** | ng/g | <2 | <2 | <2 | <2 | <2 | <2 | <2 |
| **Dibenzo[a.e]pyrene** | ng/g | <2 | <2 | <2 | <2 | <2 | <2 | <2 |
| **Dibenzo[a.l]pyrene** | ng/g | <2 | <2 | <2 | <2 | <2 | <2 | <2 |
| **Dibenzo[a.h]pyrene** | ng/g | <2 | <2 | <2 | <2 | <2 | <2 | <2 |
| **Σ PAHs** | **ng/g** | **49** | **78** | **76** | **81** | **76** | **83** | **42** |
| **HCB, TBT, hydrocarbons** | | | | | | | | |
|  | **Units** | **T0** | **Site I** | **Site II** | **Site III** | **Site IV** | **Site V** | **Site VI** |
| **Hexachlorobenzene (HCB)** | mg/Kg | <0.02 | <0.02 | <0.02 | <0.02 | <0.02 | <0.02 | <0.02 |
|  |  |  |  |  |  |  |  |  |
| **Tributiltin (as Sn)** | µg/Kg | <20 | <20 | <20 | <20 | <20 | <20 | <20 |
|  |  |  |  |  |  |  |  |  |
| **Light hydrocarbons ≤ C12** | mg/Kg | <10 | <10 | <10 | <10 | <10 | <10 | <10 |
| **Heavy hydrocarbons > C12** | mg/Kg | <10 | <10 | <10 | <10 | <10 | <10 | <10 |

**Table S4: Pairwise Adonis values on Unweighted and Weighted Unifrac distances at different time-points (A) and among different sites for each time-point (B). Only adjusted p-values lower than 0.05 were considered as significant.** **Data collected from a total of 10 biological replicates for each site/sampling time were considered.**

**A)**

| **Metrics** | **pairs** | **Df** | **SumsOfSqs** | **F.Model** | **R2** | **p.value** | **p.adjusted** | **sig** |
| --- | --- | --- | --- | --- | --- | --- | --- | --- |
| Unweighted Unifrac | Day_14 vs Day_0 | 1 | 1.066 | 5.043 | 0.069 | 0.001 | 0.003 | * |
| Unweighted Unifrac | Day_14 vs Day_3 | 1 | 2.098 | 10.533 | 0.0826 | 0.001 | 0.003 | * |
| Unweighted Unifrac | Day_0 vs Day_3 | 1 | 0.816 | 4.27 | 0.059 | 0.001 | 0.003 | * |
| Weighted Unifrac | Day_14 vs Day_0 | 1 | 0.196 | 2.675 | 0.0378 | 0.026 | 0.078 |  |
| Weighted Unifrac | Day_14 vs Day_3 | 1 | 0.121 | 1.868 | 0.015 | 0.1 | 0.3 |  |
| Weighted Unifrac | Day_0 vs Day_3 | 1 | 0.227 | 3.677 | 0.052 | 0.007 | 0.021 | . |

**B)**

| **Day** | **Metrics** | **pairs** | **Df** | **SumsOfSqs** | **F.Model** | **R2** | **p.adjusted** |
| --- | --- | --- | --- | --- | --- | --- | --- |
| Day3 | Unweighted Unifrac | T0 vs I | 1 | 0.486410654 | 2.37033273 | 0.116362 | 0.126 |
| Day3 | Unweighted Unifrac | T0 vs II | 1 | 0.734604765 | 3.83883162 | 0.17578 | 0.021 |
| Day3 | Unweighted Unifrac | T0 vs III | 1 | 0.621296622 | 3.514066996 | 0.163338 | 0.021 |
| Day3 | Unweighted Unifrac | T0 vs IV | 1 | 0.589985338 | 3.117330954 | 0.14762 | 0.021 |
| Day3 | Unweighted Unifrac | T0 vs V | 1 | 0.610842976 | 3.039858835 | 0.151691 | 0.021 |
| Day3 | Unweighted Unifrac | T0 vs VI | 1 | 0.706075309 | 3.612113594 | 0.167134 | 0.021 |
| Day3 | Unweighted Unifrac | I vs II | 1 | 0.28370837 | 1.561312102 | 0.079816 | 1 |
| Day3 | Unweighted Unifrac | I vs III | 1 | 0.278036397 | 1.66336787 | 0.084592 | 0.798 |
| Day3 | Unweighted Unifrac | I vs IV | 1 | 0.327575962 | 1.823820862 | 0.092001 | 0.525 |
| Day3 | Unweighted Unifrac | I vs V | 1 | 0.306197127 | 1.605422449 | 0.086288 | 1 |
| Day3 | Unweighted Unifrac | I vs VI | 1 | 0.455486591 | 2.451168911 | 0.119855 | 0.126 |
| Day3 | Unweighted Unifrac | II vs III | 1 | 0.185405899 | 1.209380739 | 0.062958 | 1 |
| Day3 | Unweighted Unifrac | II vs IV | 1 | 0.345323977 | 2.083232649 | 0.10373 | 0.168 |
| Day3 | Unweighted Unifrac | II vs V | 1 | 0.297570403 | 1.690105807 | 0.090428 | 0.756 |
| Day3 | Unweighted Unifrac | II vs VI | 1 | 0.550928341 | 3.203480902 | 0.151083 | 0.021 |
| Day3 | Unweighted Unifrac | III vs IV | 1 | 0.283719133 | 1.876391015 | 0.094403 | 0.294 |
| Day3 | Unweighted Unifrac | III vs V | 1 | 0.334793368 | 2.083980479 | 0.109201 | 0.063 |
| Day3 | Unweighted Unifrac | III vs VI | 1 | 0.563201973 | 3.577720788 | 0.165806 | 0.021 |
| Day3 | Unweighted Unifrac | IV vs V | 1 | 0.227949583 | 1.311255361 | 0.071609 | 1 |
| Day3 | Unweighted Unifrac | IV vs VI | 1 | 0.537291045 | 3.162837876 | 0.149452 | 0.021 |
| Day3 | Unweighted Unifrac | V vs VI | 1 | 0.436408015 | 2.418834319 | 0.124561 | 0.168 |
| Day14 | Unweighted Unifrac | III vs IV | 1 | 0.409991141 | 2.213687864 | 0.109514 | 0.084 |
| Day14 | Unweighted Unifrac | III vs V | 1 | 0.324239431 | 1.800764089 | 0.090944 | 0.378 |
| Day14 | Unweighted Unifrac | III vs VI | 1 | 0.596007265 | 3.058053479 | 0.14522 | 0.042 |
| Day14 | Unweighted Unifrac | III vs II | 1 | 0.246582487 | 1.39457262 | 0.071905 | 1 |
| Day14 | Unweighted Unifrac | III vs T0 | 1 | 1.00773342 | 5.204305345 | 0.224282 | 0.021 |
| Day14 | Unweighted Unifrac | III vs I | 1 | 0.556134423 | 3.163961385 | 0.149498 | 0.021 |
| Day14 | Unweighted Unifrac | IV vs V | 1 | 0.276374617 | 1.433087767 | 0.073745 | 1 |
| Day14 | Unweighted Unifrac | IV vs VI | 1 | 0.498526021 | 2.40029611 | 0.11766 | 0.021 |
| Day14 | Unweighted Unifrac | IV vs II | 1 | 0.410538175 | 2.165151367 | 0.107371 | 0.084 |
| Day14 | Unweighted Unifrac | IV vs T0 | 1 | 0.923864564 | 4.475426123 | 0.199125 | 0.021 |
| Day14 | Unweighted Unifrac | IV vs I | 1 | 0.701967253 | 3.722630877 | 0.171371 | 0.021 |
| Day14 | Unweighted Unifrac | V vs VI | 1 | 0.405307123 | 2.001093321 | 0.100049 | 0.273 |
| Day14 | Unweighted Unifrac | V vs II | 1 | 0.331511811 | 1.797191336 | 0.09078 | 0.336 |
| Day14 | Unweighted Unifrac | V vs T0 | 1 | 0.880319145 | 4.373608694 | 0.195481 | 0.021 |
| Day14 | Unweighted Unifrac | V vs I | 1 | 0.543586391 | 2.963667338 | 0.141372 | 0.021 |
| Day14 | Unweighted Unifrac | VI vs II | 1 | 0.465796687 | 2.337139289 | 0.11492 | 0.042 |
| Day14 | Unweighted Unifrac | VI vs T0 | 1 | 0.680033973 | 3.146545347 | 0.148797 | 0.021 |
| Day14 | Unweighted Unifrac | VI vs I | 1 | 0.450665229 | 2.273127199 | 0.112125 | 0.105 |
| Day14 | Unweighted Unifrac | II vs T0 | 1 | 0.72583822 | 3.665126935 | 0.169172 | 0.042 |
| Day14 | Unweighted Unifrac | II vs I | 1 | 0.414011363 | 2.297815995 | 0.113205 | 0.063 |
| Day14 | Unweighted Unifrac | T0 vs I | 1 | 0.61672403 | 3.130661654 | 0.148157 | 0.021 |

***Figure S1. Pearson’s correlation between the coordinates along the first component of variation of the sediment’s PCA and of the gene expression’s PCA.***


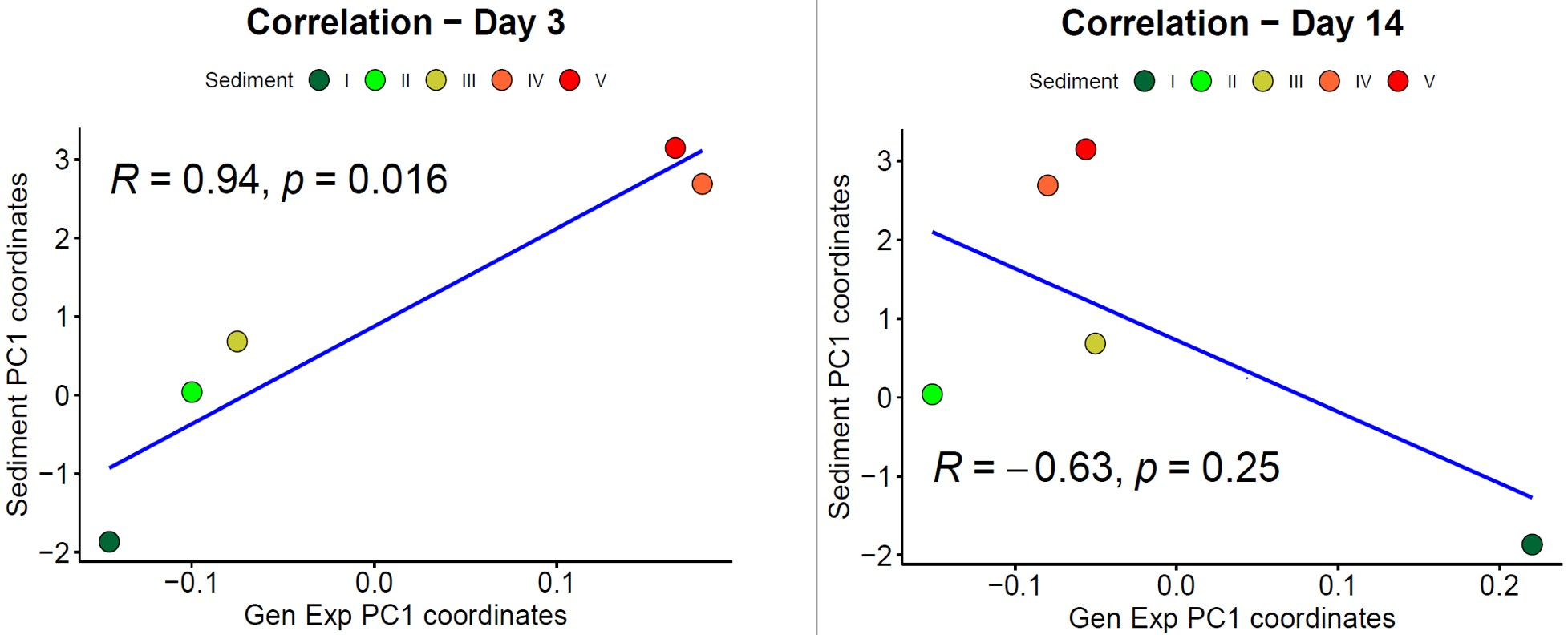


***Figure S2. Principal Coordinate Analysis (PcoA) plot using Unweighted and Weighted UniFrac dissimilarities (ASV level) of the digestive gland and sediments microbiota.*** *A) PCoA with Unweighted and Weighted UniFrac considering the microbial composition of digestive gland microbiota for the three time-points (yellow: Day 0; orange: Day 3; light blue: Day 14); B) PCoA on digestive gland microbiota of Manila clam exposed to different sediments using Unweighted UniFrac distance and split for collection date (i.e., Day 3 and Day 14) . Sites are indicated by different colours; C) PCoA using Unweighted and Weighted UniFrac distances based on sediments microbiota. P values reported in the two plots are the results of Adonis test. Sites are identified by different colours.*

**A)
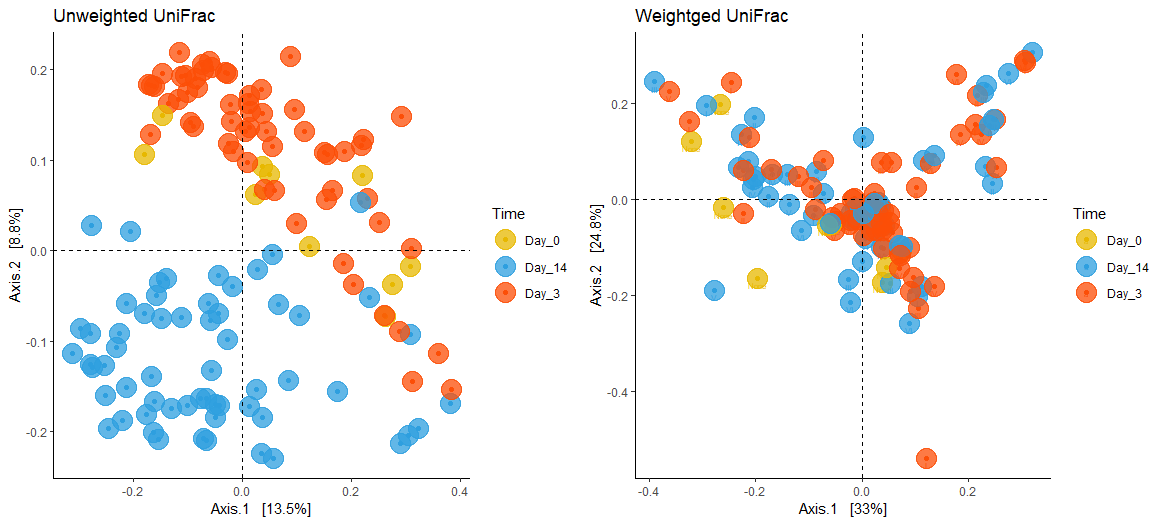
**

**B)**

**
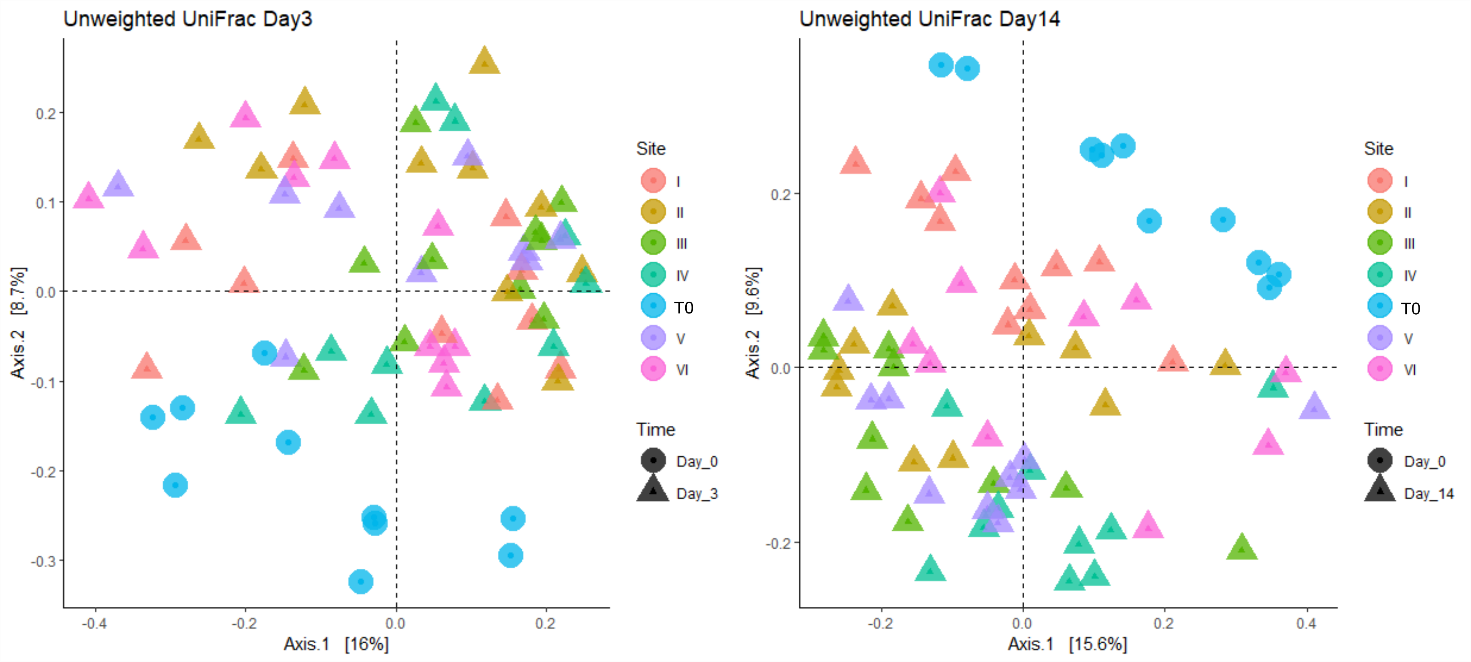
**

**C)**

**
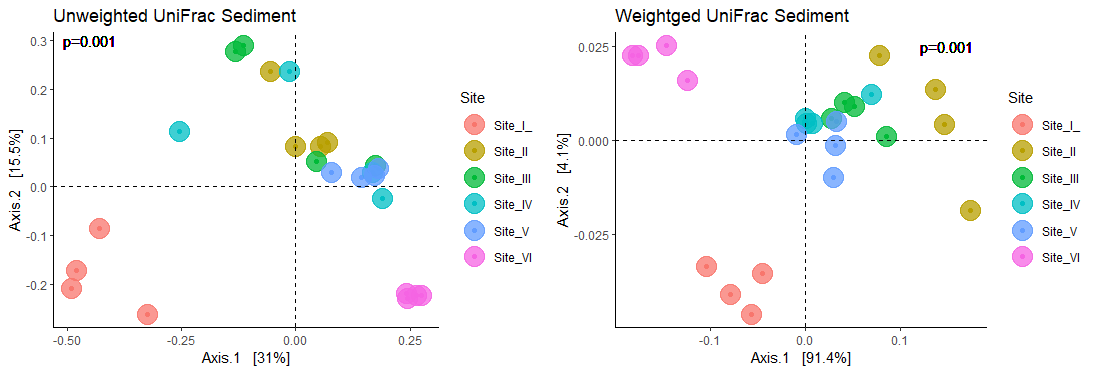
**

***Figure S3. DESeq2 results by collection date****. To highlight the phylogenetic difference showed by FigS2A, DESeq2 was applied between Day0 and Day14 (A) and between Day 3 and Day 14 (B). Red-line indicates the p-value threshold of 0.05. C) As reported by Unweighted UniFrac, Day 14 was particularly enriched in ASV belonging to the Firmicutes phylum.*

**
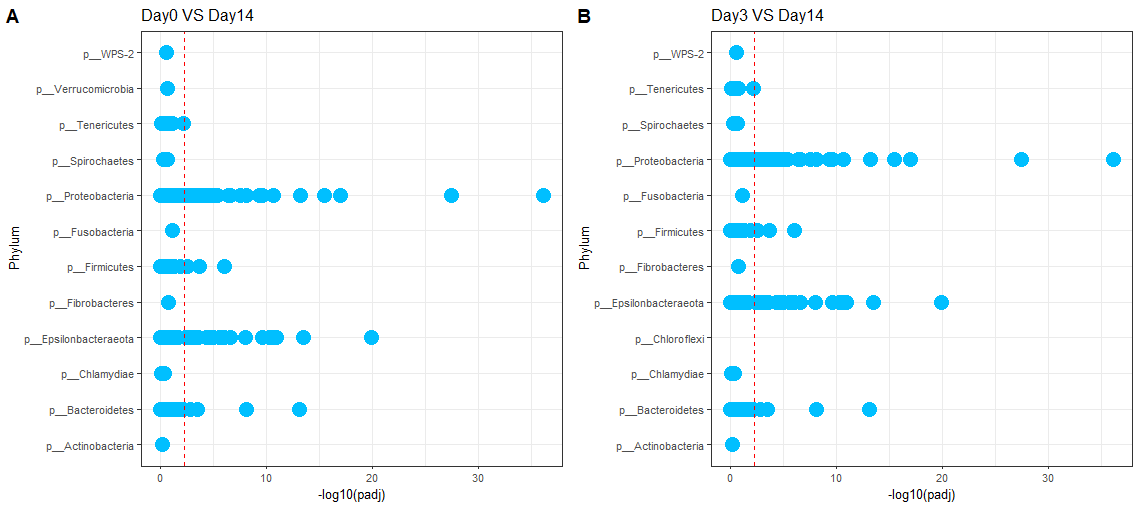
**

**C**

**
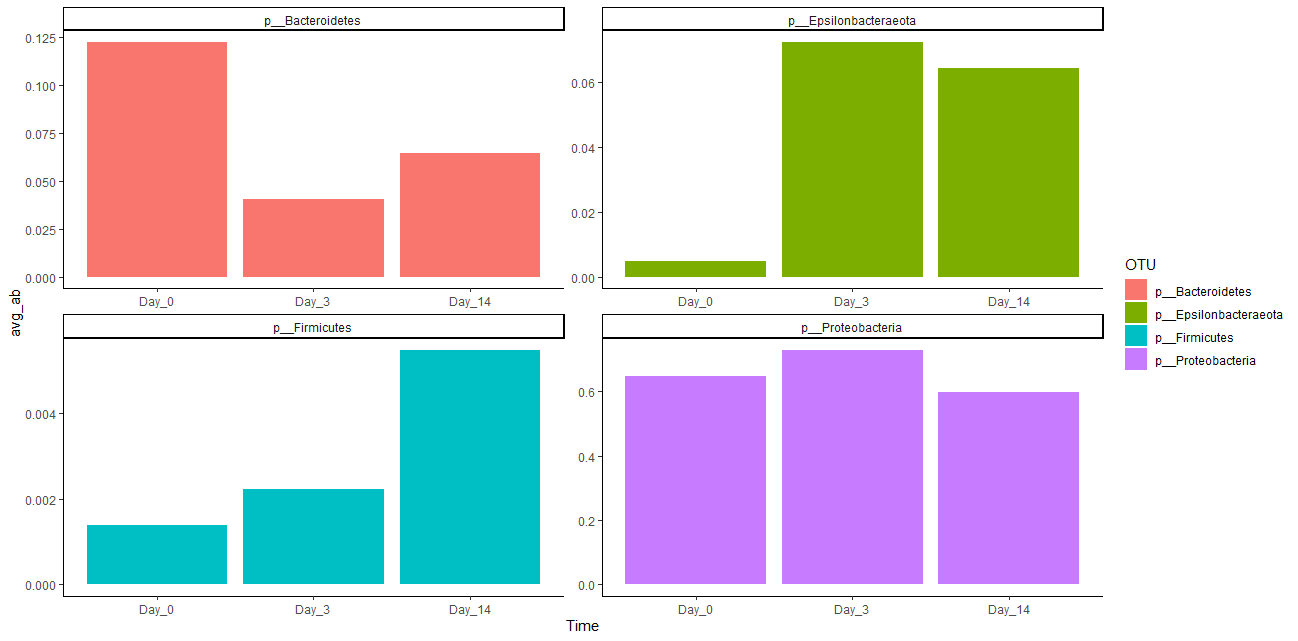
**

***Figure S4. Alpha diversity (Shannon index, Simpson index and Richness) obtained considering Manila clam microbiota and sediments****. For Manila clam microbiota diversity indexes were obtained considering different sampling time (T0, Day3 and Day14) and different sites within each sampling time (Day 3 and Day 14).*

**
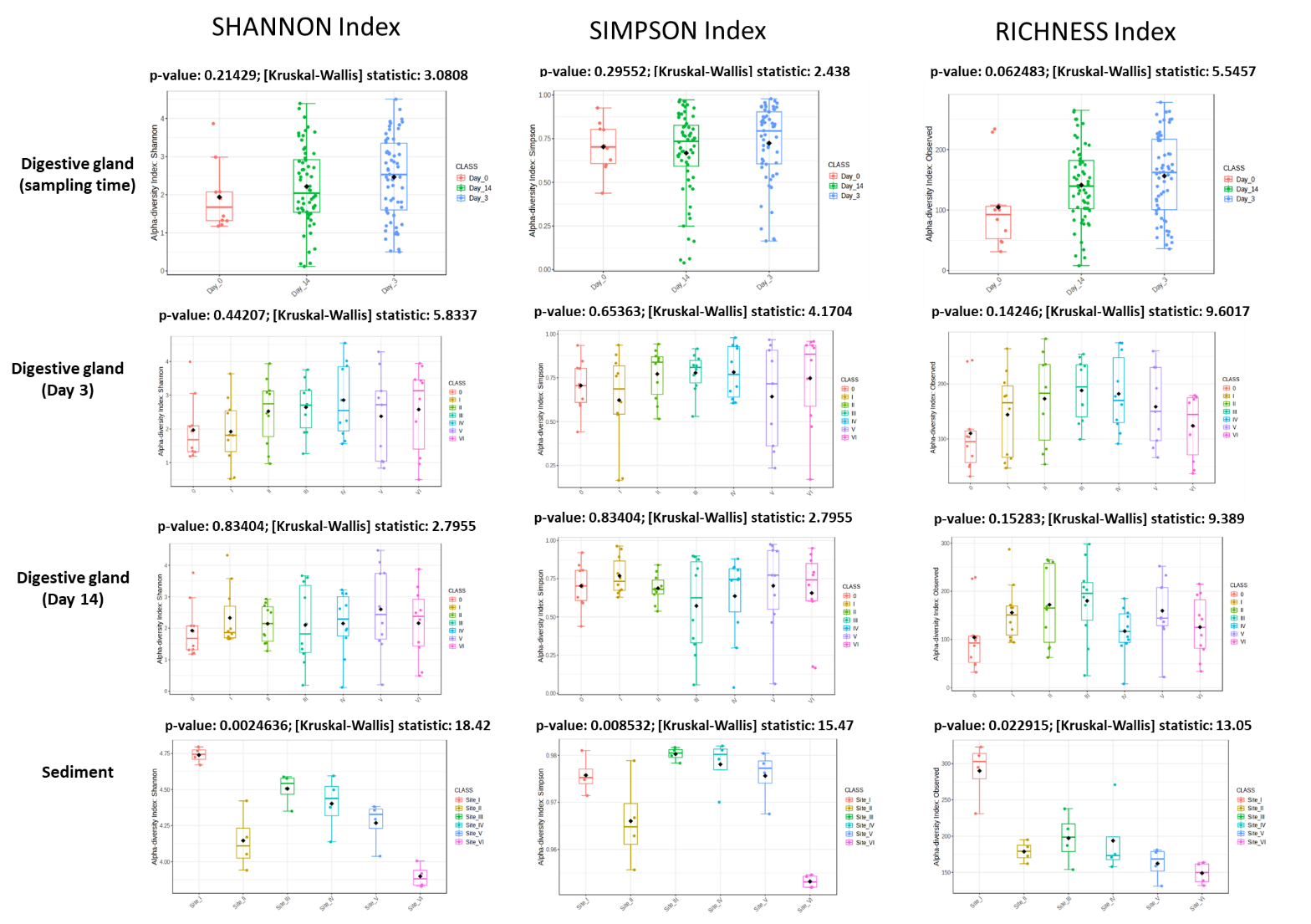
**

***Figure S5.*** *Barplot representing the number of unique ASVs for each bacteria Phylum in group I and group II, A and B respectively.*

**
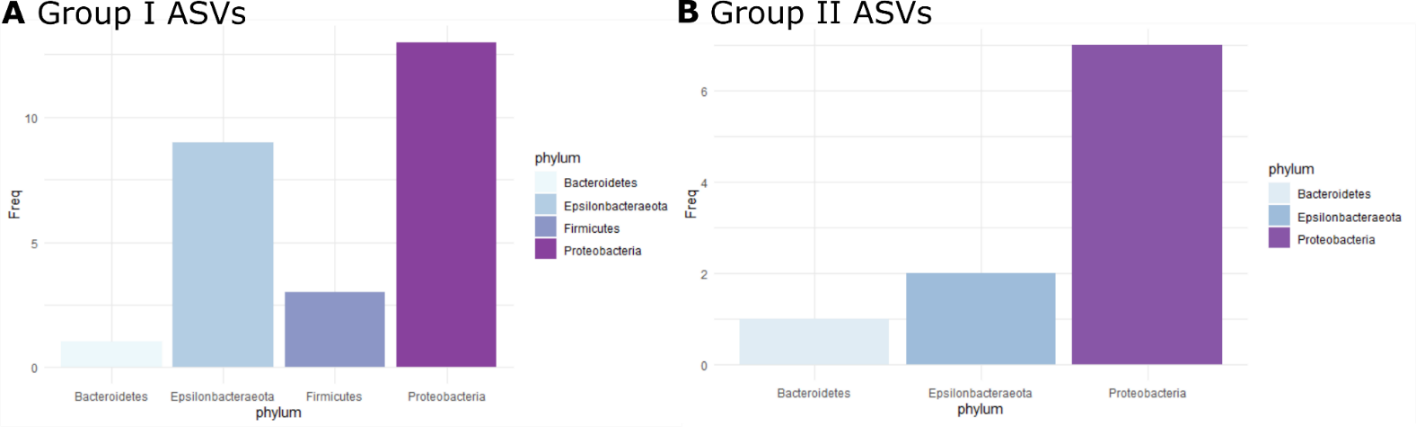
**

***Figure S6. Changes in relative abundance of the significant ASVs for the two groups and for every site considered****: Site I (A); Site II (B,C); Site III (D,E); Site IV (F,G); Site V (H,I); Site VI (L,M). Green and Red lines identify ASVs belonging to group I and II, respectively.*

**
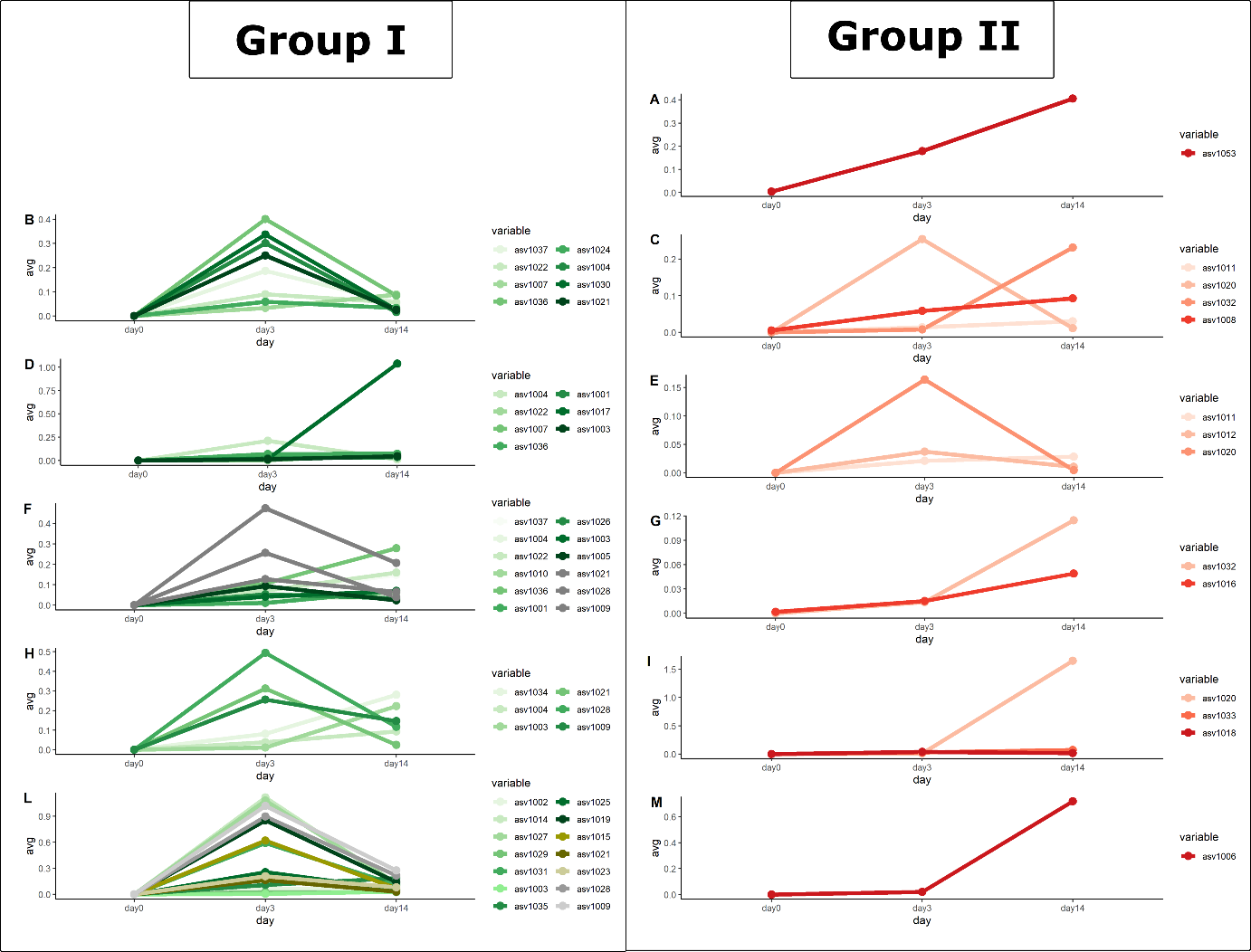
**
